# Habituation to pain: self-report, electroencephalography, and functional magnetic resonance imaging in healthy individuals. A scoping review and future recommendations

**DOI:** 10.1097/j.pain.0000000000003052

**Published:** 2023-10-18

**Authors:** Maite M. van der Miesen, Elbert A. Joosten, Amanda L. Kaas, David E.J. Linden, Judith C. Peters, Catherine J. Vossen

**Affiliations:** a Department of Anesthesiology and Pain Management, School for Mental Health and Neuroscience, Maastricht University, Maastricht, the Netherlands; b Department of Anesthesiology and Pain Medicine, Maastricht University Medical Centre, Maastricht, the Netherlands; c Department of Cognitive Neuroscience, Maastricht University, Maastricht, the Netherlands; d Department of Psychiatry and Neuropsychology, School for Mental Health and Neuroscience, Maastricht University, Maastricht, the Netherlands

**Keywords:** Habituation, Sensitization, Pain, Individual differences, Learning

## Abstract

Habituation to pain is a fundamental learning process and important adaption. Yet, a comprehensive review of the current state of the field is lacking. Through a systematic search, 63 studies were included. Results address habituation to pain in healthy individuals based on self-report, electroencephalography, or functional magnetic resonance imaging. Our findings indicate a large variety in methods, experimental settings, and contexts, making habituation a ubiquitous phenomenon. Habituation to pain based on self-report studies shows a large influence of expectations, as well as the presence of individual differences. Furthermore, widespread neural effects, with sometimes opposing effects in self-report measures, are noted. Electroencephalography studies showed habituation of the N2-P2 amplitude, whereas functional magnetic resonance imaging studies showed decreasing activity during painful repeated stimulation in several identified brain areas (cingulate cortex and somatosensory cortices). Important considerations for the use of terminology, methodology, statistics, and individual differences are discussed. This review will aid our understanding of habituation to pain in healthy individuals and may lead the way to improving methods and designs for personalized treatment approaches in chronic pain patients.

## Abstract 2

Supplemental Digital Content is Available in the Text.

## 1. Introduction

The concept of habituation has a long history, and it is one of the first and simplest forms of learning that has been studied.^13^ Ever since the first known research description of habituation in 1887,^70^ the definition of habituation has been under construction and in development. In the context of exercise, habituation has been named “practice effect,”^33^ whereas in other fields, it was named “adaptation,” “extinction,” or “fatigue.”^13,94,95^ The first list of behavioral characteristics of habituation was composed by Thompson and Spencer.^95^ They established a definition for habituation, which was updated in 2009 by Rankin et al.^75^: “a behavioral response decrement that results from repeated stimulation and that does not involve sensory adaptation/sensory fatigue or motor fatigue.”^75^ Changes in behavioral response may be linear or exponential and show recovery over time.^75^ Although the neurobiology of habituation is still not completely understood, it became clear that it involves some form of neural plasticity.^13^ The process that early on was observed on the behavioral level as “fatigue” of the body turned out to have an underlying neural process. When merely sensory or motor fatigue is present, it is nowadays excluded from the general definition of habituation. However, some scholars adhere to 2 definitions, where central habituation is taking place at the spinal cord and brain (excluding sensory effects) and peripheral habituation has been related to effects taking place at the sensory afferent level (also referred to as “fatigue” or “adaptation”).^32,76^ Throughout this review, we adhere to habituation as the central process and refer to peripheral habituation in the discussion of sensory effects.

Short-term and long-term effects of habituation differ with respect to their timescale and have been recognized as different concepts. They are defined as response decrements within one session (with a time course over minutes up to a few hours) and over several daily repetitions (with a time course of hours, days, or weeks), respectively.^75,89^ Similarly, qualitatively different mechanisms are involved in short-term and long-term habituation.^75^ Besides habituation, repeated stimulation may instead also lead to sensitization: “an increase in behavioral response over time.”^34,101^ Sensitization is, like habituation, considered a simple form of nonassociative learning.^75,95^ Yet, it is unclear how these processes might interact.

Several theories for habituation and sensitization have been proposed.^94^ The current prominent theories are the stimulus model comparator theory, the Wagner–Konorski Gnostic Unit Theory, and the dual process theory. The stimulus model comparator theory^90^ describes that new stimuli are first amplified but inhibited when they resemble the model of stimuli (ie, a form of predictive coding). The same applies to the Wagner–Konorski Gnostic Unit Theory,^53,106^ except that this theory also involves an associative network that is able to influence the model. The dual process theory states that habituation and sensitization are independent processes that interact and result in a final response.^34^

Habituation and sensitization are central concepts for the understanding of pain. Pain is an important mechanism in signaling threat to the body, and learning is critical in handling pain. Habituation and sensitization to pain are the core part of this learning process and may result in (mal-)adaptive changes in pain processing. The underlying motivational–ethological framework poses that habituation to pain occurs when threat is relatively low and it is adaptive to pursue other goals.^15^ The environment and social context also seem to be of particular importance.^15^ The literature of habituation to pain dates back as far as the 1960s. Early studies used repeated immersions in cold water and focused mostly on physiological signals like blood pressure.^23,55^ However, pain sensation in itself became more important in understanding habituation to pain and was reported to diminish after several days of the cold pressor test.^23^ Moreover, LeBlanc and Potvin^55^ suggested the involvement of the central nervous system in habituation to pain. Subsequent studies used electrical dental stimulation,^14,26^ measured firing of trigeminal peripheral nerves,^46^ and evoked potentials^14^ to address habituation to pain in various settings. Since these early studies about habituation to pain, the literature has expanded tremendously, including standardized methods for measuring pain sensations using the numeric rating scale (NRS) and visual analogue scale (VAS), development of paradigms as well as testing in different contexts, and advances in measuring such as electroencephalography (EEG) and functional magnetic resonance imaging (fMRI) for the investigation of neural substrates of habituation. Behavioral and neural responses are now commonly seen as measures of habituation.^75^ It needs to be stressed that most literature does not distinguish between habituation of nociceptive processes or pain.^1^ There is a conceptual difference between nociceptive processes and pain. Pain refers to an unpleasant sensory and emotional experience, whereas nociception describes the processing of noxious stimuli in the nervous system.^74^ Neural activity described in fMRI and EEG literature may or may not result in (self-report of) pain perception.^1^ Although for this review, we adhere to descriptions as used in the original articles, this is an important point to consider for fMRI and EEG studies that do not include self-report. So far, the literature on habituation to pain including EEG and fMRI measures have not been summarized. This is of major importance to increase our understanding of the neural processes underlying habituation to pain. Furthermore, the link between neural and behavioral measures of habituation is not well understood. For example, standard measures, such as self-report ratings, have only recently been explored in conjunction with potential neural correlates. Therefore, the objective of this scoping review is to summarize the current state of the field of habituation to pain using self-report ratings, EEG, and fMRI in healthy individuals. To limit the scope of the review, behavioral measures, such as reflexes, were not considered. Moreover, recommendations for further studies are provided, including the use of terminology, experimental design, and methods to analyze habituation to pain.

## 2. Methods

This scoping review follows the PRISMA Extension for Scoping Reviews (PRISMA-ScR) guidelines and reports accordingly.^98^ The review protocol is preregistered at the Open Science Framework: osf.io/nypbw.

### 2.1. Search strategy

PubMed, PsycINFO, and Web of Science databases were searched for eligible studies from database initiation until July 2020. Search terms consisted of elements related to pain AND habituation (the search strategy for PubMed can be found in the Supplementary Files, available at http://links.lww.com/PAIN/B920). Terms related to “repeated painful stimulation” were also added, to ensure a full inclusion of studies where habituation might be investigated. No filters were applied. Reference lists of articles fulfilling the inclusion criteria were manually screened for additional references. Before finalizing the article, a final search was performed including all articles published until 20 August 2022.

### 2.2. Study criteria

Exclusion criteria were preregistered and evaluated in order (see Table 1; inclusion criteria are added for clarity). This review focuses on self-report ratings and brain measures (EEG and fMRI) of habituation. Consequently, articles should include one of these 3 measures and describe a habituation process involving repeated painful stimulation at the same intensity. This latter point was evaluated in 2 ways: first, the concept of habituation should be discussed in the article and, second, a change in pain, measured through ratings or neural activity, should be quantified. The decision whether a change in pain, measured through ratings or neural activity, is referred to as habituation or not was left to the original authors. With respect to intervention studies, we only included intervention studies if they obtained measures of habituation separately before and after the intervention (ie, to see the effect of an intervention on habituation). Studies that merely reported a change in pain ratings (or amplitude) after an intervention were excluded. Habituation effects of an intervention that itself included repeated stimulation (eg, transcutaneous electrical nerve stimulation) were also excluded. Accordingly, we only included effects of repeated stimulation during control conditions of interventional studies if habituation was an outcome measure of the interventional study. Because of our broad search, many studies were identified with well-known paradigms (eg, cold pressor test, high-frequency stimulation), but either these studies did not discuss their results in the context of habituation or they did not quantify habituation before or after an intervention. Therefore, these studies were not included. Studies that included paradigms where the stimulation intensity was adapted as a measure of habituation were also not included. Studies on habituation of pain thresholds were also excluded because they do not require repeated stimulation above the pain threshold, which is our primary interest and involves a different evaluation process (eg, decision making whether something is painful and how much pain can be tolerated). Articles were screened during selection whether the stimulus was painful, through the use of calibration, description of the stimuli as painful (eg, stimuli elicited a sharp painful sensation), or the consistent use of habituation to pain throughout the article.

**Table 1:** Inclusion and exclusion criteria

Criterion	Included	Excluded
1. Article type	Research manuscripts	Review, commentary, editorial, thesis, book section, meta-analyses
2. Population	Adults	Animals, children
3. Paradigm	Painful repeated stimulation related to habituation	No painful stimuli, visual stimuli, auditory stimuli, unrelated paradigm (eg, survey, sensitization)
4. Response measurement	Multiple pain ratings or neural activity	Measures of behavioral habituation, blood pressure, pain threshold, reflex, skin conductance
5. Language	English	Other
6. Imaging	EEG and fMRI	Studies without functional imaging during the habituation process and PET, sMRI, fNIRS, DTI
7. Intervention	Factors influencing habituation	Habituation effects of interventions, for example, electrical nerve stimulation, spinal cord stimulation
8. Other		Intracranial recordings, n = 1, full text not available

DTI, diffusion tensor imaging; EEG, electroencephalography; fMRI, functional magnetic resonance; fNIRS, functional near-infrared spectroscopy; PET, positron emission tomography; sMRI, structural magnetic resonance imaging.

### 2.3. Selection procedure

References from PubMed, PsycINFO, and Web of Science databases were combined in EndNote and deduplicated. Titles and abstracts were screened for eligibility by 2 independent reviewers (M.vdM. and C.V.). Full-text screening was performed as a final selection round for all articles that were likely relevant or unclear based on the title and abstract only. This was performed by the same reviewers (M.vdM. and C.V.). Discrepancies for inclusion were discussed with a third independent reviewer (J.P.) to come to an agreement. This review was originally preregistered at the Open Science Framework to include both healthy participants and chronic pain patients, but it was decided to deviate from the protocol and only include healthy participants. Our decision was made based on the fact that there is abundant literature available to describe the current state and neural mechanisms in healthy participants. This review will serve as an important step and foundation for further review of habituation to pain in chronic pain patients.^102^

### 2.4. Data extraction and synthesis

All relevant data from the texts were extracted by the first reviewer (M.vdM.). Extracted data contained study design, description of population, period (long-term or short-term habituation), habituation protocol, type of pain stimuli, site and number of pain stimuli, duration, interstimulus interval, statistical analysis, brain activity (eg, event-related potentials, activated areas), and variables that might affect habituation (eg, anxiety). Studies were categorized based on measurement type (ie, self-report, EEG and fMRI) and topic (eg, experimental settings).

### 2.5. Visualization

Extracted data were visualized using R Statistical Software 4.2.1^73^ in RStudio version 2022.7.1.554^82^ using packages *ggplot2* 3.3.6,^108^ *ggforce* 0.3.4,^71^ and *ggpubr* 0.4.0.^48^ To visualize areas that show fMRI activity related to habituation, only those articles were included that reported significant habituation effects in healthy participants and were corrected for multiple comparisons. Peak MNI coordinates were imported into BrainVoyager 22.2 (Brain Innovation, Maastricht, the Netherlands),^30^ and a sphere (5-mm radius) was drawn around the peak coordinates followed by the export of these regions of interest. This approach facilitates the visualization of the individual study peak coordinates and consistency in the peak localization of habituation effects, rather than merely the overlap of clusters. Talairach coordinates were converted to MNI coordinates using BioImage Suite Web.^54,67^ The regions of interest were then visualized using MRICron.^80^

## 3. Results

**Figure 1.**
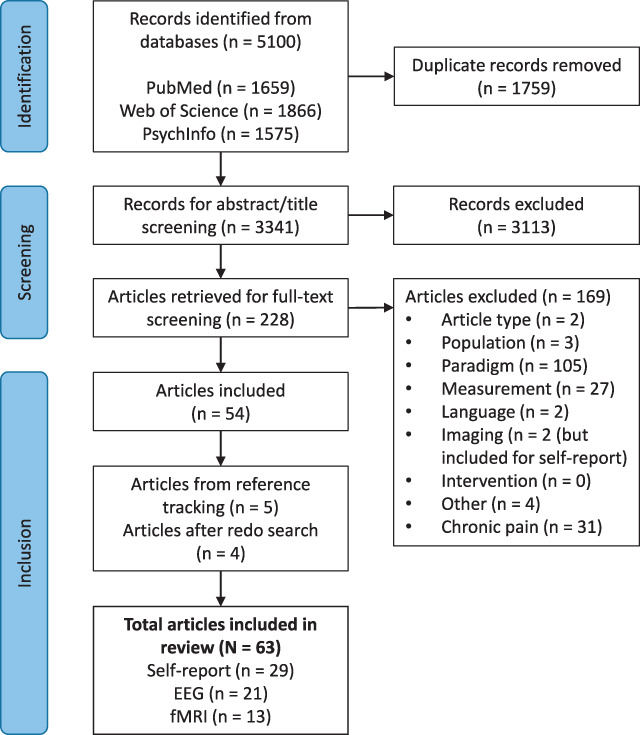
Flow diagram of selection and inclusion.

**Figure 2.**
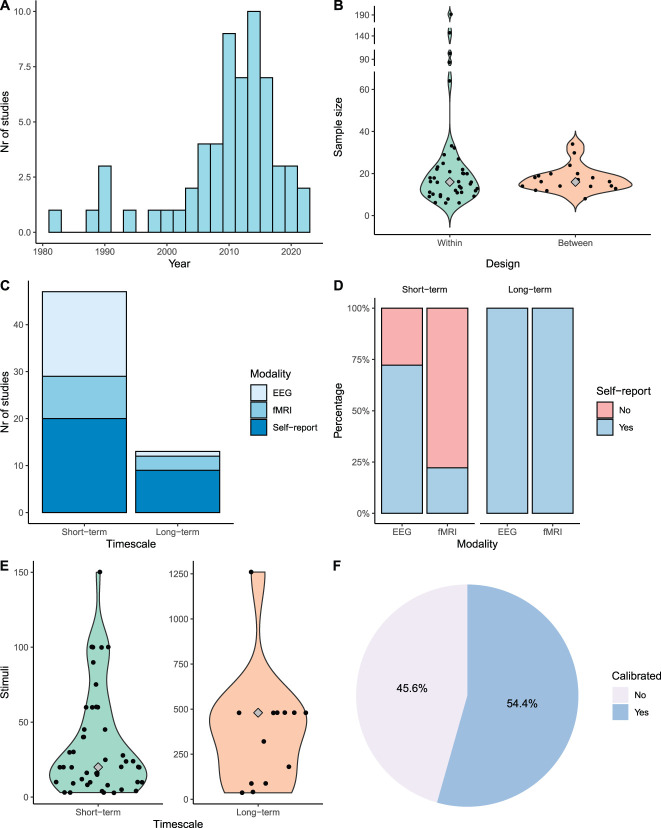
Methodological variations and descriptives of the studies included. Grey diamond indicates the median. (A) Number of studies per publication year. (B) Sample size for within-subject and between-subject design. (C) Number of studies per modality and timescale. (D) Percentage of studies including self-report. (E) Number of stimuli to measure habituation per timescale. (F) Use of individual calibration in studies. Note: this visualization is based on a total of 60 studies because the same data were included in 3 studies.

Database search of PubMed, PsycINFO, and Web of Science identified 5100 records (Fig. 1). Automatic and manual deduplication allowed to remove 1759 records, resulting in 3341 records for abstract and title screening of which 228 articles were retrieved for full-text screening. After screening by 2 independent reviewers (M.vdM. and C.V.) and discussing discrepancies with a third independent reviewer (J.P.), 54 articles were selected and included. Reference list tracking added another 5 articles, and a final repetition of the searches to ensure that no recent articles were missed allowed to add 4 more articles, resulting in a total of 63 articles. Two studies that did not meet the imaging criterion were included for the self-report results only. As described in the methods section, articles including a chronic pain population were excluded. Currently, there is a movement toward open science and data sharing. However, in our studies, only one study was preregistered,^93^ and 2 studies shared data and/or code.^6,59^ The majority of the articles included in this review are more than 5 years old (Fig. 2A), and this likely contributed to the limited open science practices. Furthermore, the sample sizes are, in general, small, with a median sample of n = 16 for within-subject and between-subject studies (Fig. 2B). Studies with larger sample sizes are needed to increase power and chance of replicability, but only a few of such studies were identified. Three different modalities were included: self-report (n = 29), EEG (n = 21), and fMRI (n = 13). Most studies investigated short-term habituation with self-report and/or EEG (Fig. 2C). All long-term studies using EEG or fMRI (n = 4) also included self-report, whereas for short-term studies, this was only included in 2 fMRI studies (22.2%) and in 13 EEG studies (72.2%) (see Figure 2D) (note that these percentages are in agreement with Fig. 2, where articles using the same data sets are omitted).

### 3.1. Methodological variations

In terms of measuring habituation, there have been considerable methodological variations (see Fig. 2). First, the duration of the study and measured habituation effect does considerably differ. Long-term studies (n = 14) measured habituation over several days, whereas short-term studies (n = 49) defined habituation based on a single session. Long-term paradigms included 8 to 11 days in which participants received several blocks of painful (heat) stimulation on each day (a procedure initially developed by Bingel et al.^9^). Changes in self-report or brain activity were then measured over days and sometimes as well within a day. Short-term paradigms showed more variations in setup, time window, and the number of stimuli (given the short timescale), ranging from only 3 to 150 stimuli (median = 20; Fig. 2E). In addition, a wide range of stimuli durations have been used from only 0.1 ms (electrical stimuli) or 1 ms (heat stimuli using a laser) to 40 seconds (heat stimuli using a thermode) or even minutes in the case of a cold pressor test. Second, studies differed in whether they used individual calibration to determine the strength of the stimulus (Fig. 2F). When calibration is not used, it may result in large differences in experienced pain and ceiling or floor effects (eg, the stimuli might not be that painful for some participants, whereas others might perceive them as too painful). This difference in calibration to determine the strength of the stimulus may affect the outcome (ie, habituation or sensitization patterns) because habituation tends to be more rapid and/or more pronounced with lower stimulus intensities.^75^ Third, changes in pain intensity were measured in various ways, for example, within a run (ie, series) of stimuli and/or across runs that could be spaced within or across days. Such variations in timescales and range of stimuli result in different operationalizations of habituation (ie, how to define and measure habituation), hampering comparisons across studies. Fourth, various methods were used to define and present habituation. For example, percentage change from a last to first block of stimulation has been reported, but direct comparison of trials or testing for linear trends in the data was also reported. With methodological variations to define habituation, utmost care should be taken for a correct interpretation and comparison. Averaging over trials of the dependent variable can lead to different results than the “raw” single trial or block data, which is preferred when the data quality allows this. The variation in “calculating” and defining habituation is closely linked to a fifth issue, which is the use and selection of the statistical model. All the methodological variations result in difficulties to correctly compare the outcome of the individual studies. In this context, the formulation of general conclusions is challenging. The next sections describe the results of studies on habituation to pain in healthy participants as related to the modality self-report (section 3.2), EEG (section 3.3), and fMRI (section 3.4) as well as associations among them (section 3.5).

### 3.2. Habituation to pain: self-report

#### 3.2.1. Type of stimuli

**Figure 3.**
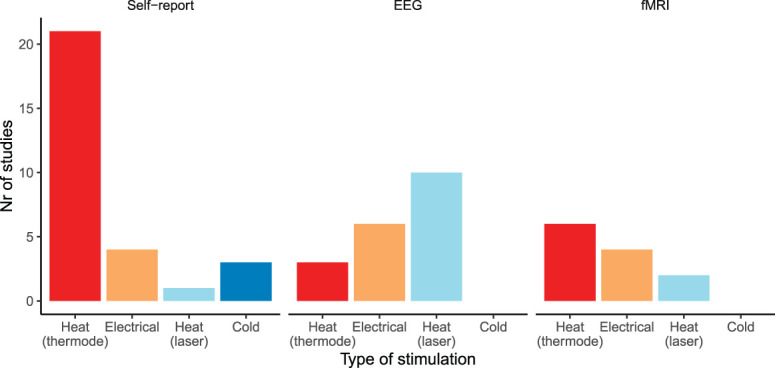
Type of stimulation used in studies on habituation to pain and evaluated based on self-report (left), EEG (middle), or fMRI (right).

The majority of self-report habituation studies in healthy participants have used heat stimuli using a thermode (n = 21), (Fig. 3). Several studies using heat stimulation in long-term paradigms showed habituation over days (Tables 2 and 4).^9,20,76,77,92^ Some of these long-term studies reported sensitization patterns within one day.^11,25^ When investigating short-term patterns, however, which should be equivalent to effects within one day, habituation is reported as well.^52,97^ Thus, there does not seem to be a reliable pattern for these short-term effects of heat stimuli. Habituation using repeated cold (water) stimuli is also investigated (Table 2), and it was shown that some participants showed habituation over series of the cold pressor test.^111^ The latter was confirmed and extended at the group level^91^ and with the use of cold pain stimuli through an MRI-compatible thermode.^109^

**Table 2:** Habituation to pain: self-report.

	Sample size	Timescale	Type of stimuli	Site	Nr of stimuli for habituation analysis	Duration	ISI	Habituation measurement	Habituation analysis	Main habituation result
Arntz & Lousberg, 1990	Control = 14 Early = 14 Late = 14	Short term	Electrical	Right ankle	20	6 seconds	15-45 seconds	VAS: single trial	Trend over time/comparing trials directly	Decrease in experienced pain. Dishabituation after late unpredictable trial, not after early
Arntz & Van den Hout, 1988	Control = 9 Experimental = 10	Short-term	Electrical	Middle finger of non-dominant hand	10	0.5 seconds	Self-paced	VAS: single trial	Last vs first trial	No significant decrease in ratings
Arntz et al., 1991	Low anxiety, attention = 13, low anxiety/distraction = 14, high anxiety/attention = 15, high anxiety/distraction = 13	Short term	Electrical	Ankle	20	6 seconds	21-59 seconds	VAS: single trial	Trend over blocks of 4 trials	Anxiety did not influence habituation, and distraction facilitated habituation as opposed to attention
Breimhorst et al., 2012	Men = 14, women = 14	Long term (8 days)	Heat (thermode)	Left volar forearm	480	4 seconds (plateau)	0 seconds	VAS: mean per block	Trend over time	Pain intensity and pain unpleasantness decreased over days. No interaction with sex
Defrin et al., 2008	N = 18 and N = 6	Short term	Heat (thermode)	Lateral side of the lower leg	16	2 seconds	22 seconds	VAS: single trial	Trend over time	Decrease in pain, the amount depended on probe configuration and initial pain intensity. Women showed more habituation
Doganci et al., 2011	Habituation = 10, sensitization = 8, stable = 9, control = 10	Long term	Heat (thermode)	Left volar forearm	480	4 seconds (plateau)	0 seconds	VAS: average of 6 trials	Trend over days	Habituation and control groups showed decreased pain over time, whereas this was not the case for sensitization and stable group
Edwards & Fillingim, 2001	Younger = 34, older = 34	Short term	Heat (thermode)	Left volar forearm	10	0.5 seconds	2.5 seconds	VRS: single trial	Trend over time	Reduced habituation for older group at 47°C and 50°C, for both intensity and unpleasantness
Edwards et al., 2006	High catastrophizing = 18, low catastrophizing = 20	Short term	Heat (thermode)	Left dorsal forearm	10	0.5 seconds	2-3 s	VRS: single trial	Trend over time	Reduced habituation for group with higher levels of pain-related catastrophizing at 47°C and 50°C
Gács et al., 2017	N = 20	Short term	Heat (thermode)	Dominant forearm	4	20 seconds	60 seconds	VAS: single trial	Trend over trials	Higher pain for imagined vs physical pain, with sensitization for imagined pain and habituation for physical pain
Gallez et al., 2005	N = 11 and N = 9	Long term (5 days)	Heat (thermode)	Volar surface of the forearm	320	4 seconds (plateau)	—	VAS: end of each block	Mean rating over days	Decrease in pain intensity and pain unpleasantness. No generalization to other forearm
Hashmi & Davis, 2009	Men = 16, women = 16	Short term	Heat (thermode)	Dorsum of the foot	3	30 seconds	60 seconds	VAS: continuous ratings	Trend over time in total pain	Women show more habituation than men
Hashmi & Davis, 2010	Men = 19, women = 21	Short term	Heat (thermode)	Dorsum of the foot	3	30 seconds	60 seconds	VAS: continuous ratings	Trend over time in total pain	More habituation with lower temperatures (<46°C), no sex differences in habituation
Jepma et al., 2014	N = 100	Short term	Heat (thermode)	Left volar forearm	24	11 seconds (7.5 plateau)	20 seconds	VAS: single trial	Trend over time	Sensitization within runs, habituation across runs. Women show more habituation
Kleinböhl et al., 2006	N = 10	Short term	Heat (laser)	Left hand	15	100 ms	5/1.6/0.55 seconds (dependent on condition)	VAS: first and last pulse	Trend over time	Increase in pain over pulses and decrease of pain over trials, stable over runs
Koenen et al., 2017	N = 22	Short term	Heat (thermode)	Left ventral forearm	10	30 seconds	46 - 52 seconds	VAS: single trial	Trend over time	Pain intensity decreased over time
Maeoka et al., 2015	N = 8	Long term (3 days)	Heat (thermode)	Medial side of the non-dominant forearm	180	6 seconds	5 seconds	VAS: mean per block	Trend over time	Decrease for pain intensity but not unpleasantness
Quiton & Greenspan, 2008	N = 64	Long term (4 sessions)	Heat (thermode)	Right lower leg	16	15 seconds (including rising and falling)	30 seconds	VAS: single trial	Trend within and over sessions	Decrease within and over sessions for both intensities
Rennefeld et al., 2010	N = 24 (n = 12 naloxone, n = 12 control)	Long term (8 days)	Heat (thermode)	Left volar forearm	480	6 seconds	- (delay in rise and fall of temperature only)	VAS: average of block	Difference between day 1 and day 8	No effect of naloxone on pain ratings. Decreased pain over time, which was stronger for the stimulated than for the nonstimulated arm
Riedl et al., 2011	N = 13	Long term (11 days)	Heat (thermode)	Inner side of the right forearm	88	40 seconds	20 seconds	VAS: end of session	Difference between day 1 and day 11	Decrease in pain intensity (for 10 out of 13 subjects)
Rosier et al., 2002	N = 15	Long term (4 sessions)	Heat (thermode)	Ventral forearm of the nondominant hand	24	5 seconds	Unknown	VAS: single trial	Trend over sessions	No changes over sessions for all temperatures
Schmidt et al., 2015	N = 17 (arm), N = 19 (head)	Long term (8 days)	Heat (thermode)	Left volar forearm and left side of the forehead	480	6 seconds	—	VAS: mean per block	Multilevel model	Decrease over days for both groups, stronger sensitization within a day for face stimulation
Slepian et al., 2017	N = 142	Short-term	Electrical	Left leg over the sural nerve	12	25 ms (train of 5 1 ms pulses)	14-24 seconds	VAS: single trial	Multilevel model	Sensitization on the group level, large variability in slopes
Stancak et al., 1997	N = 18	Short term	Cold	Left hand	3	4 minutes	8 minutes	VAS: single trial	Trend over time	Decrease in pain intensity over cold pressor tests
Stankewitz et al., 2013	N = 27 (and 18 controls without stimulation)	Long term (11 days)	Heat (thermode)	Right forearm	88	40 seconds	120 seconds	VAS: end of block	Trend over days	No overall habituation, half of the participants showed sensitization and the other half habituation
Szikszay et al., 2022	N = 33	Short term	Heat (thermode)	Volar forearm of the nondominant hand	3	5 seconds	5, 15 or 25 seconds	VAS: continuous ratings	Comparison between the trials	Habituation effects were shown in an offset analgesia paradigm
Treister et al., 2010	N = 191	Short term	Heat (thermode)	Above thenar eminence of the left hand	5	3 seconds	12 seconds	VAS: single trial	Trend over time	Decrease in pain over 5 stimuli, no effect of sex
Weissman-Fogel et al., 2018	Sensory over-responsiveness = 14, control = 16	Short term	Heat (thermode)	Volar forearm of the dominant hand	28	0.7 seconds (peak)	8-12 seconds	VAS: single trial	Mean difference between runs	Interaction between run and group, with habituation effects for sensory overresponsiveness group only
Wilcox et al., 2015	N = 20	Short term	Cold	Thenar surface of right hand	9	16 seconds	32-36 seconds	VAS: continuous ratings	Trend over time	Decrease in pain intensity for low and high pain
Zbrozyna & Westwood, 1990	N = 14	Long term (6-10 days)	Cold	Foot	36	60 seconds	Random	VAS: single trial	Trend over time	Decrease in pain intensity over cold pressor tests for some participants

Self-report results as part of EEG or fMRI studies are included in Tables 3 and 4. The number of stimuli is reported for the habituation measurement and not necessarily referring to the total number of stimuli used in the study. If participants received 2 conditions with each 10 stimuli and habituation is calculated per condition, 10 stimuli are reported for the habituation analysis.

**Table 3:** Habituation to pain: EEG studies.

	Sample size	Timescale	Type of stimuli	Site	Nr of stimuli for habituation analysis	Duration	ISI	Habituation measurement	Habituation analysis	Main habituation result
Blom et al., 2012*	N = 16	Short term	Electrical	Left forearm	45	1, 3 or 5 2 ms pulses (interpulse interval 5 ms)	4.5-6.5 seconds	VAS: single trial in attention condition EEG: N1 and P2/P3a amplitude	Trend over blocks	Decrease in ratings and N1 and P2/P3a amplitude over time, no interaction with distraction condition
Bromm & Scharein, 1982*	N = 11	Long term (4 days)	Electrical	Left middle finger	40	20 ms train	20-40 seconds	VRS: single trial EEG: Cz amplitude	Trend over grouped stimuli within days and across days	No change in ratings and evoked potential within and across days
De Schoenmacker et al., 2022*	N = 18	Short term	Heat (thermode)	Volar forearm of the dominant hand	45	393 ms at peak	13-17 seconds	NRS: single trial EEG: N2-P2 amplitude	Trend within and across blocks	No effect over blocks for ratings and N2-P2 amplitude. Within first block a decrease in pain ratings
De Tommaso et al., 2017	Groups per age, min sample size = 20, age 18-72	Short term	Heat (laser)	Dorsum of the right hand and right supraorbital zone	60	30 ms	20-30 seconds	EEG: N2-P2 amplitude	Data divided in 3 blocks, percentage change of third compared with first block	Increase in N2-P2 habituation with age—not significant with direct comparisons between different age groups
Di Lorenzo et al., 2014	HAM = 37 HAM, LAM = 30	Short term	Electrical	Forehead	30	100 µs	14-16 seconds	EEG: N2-P2 amplitude	Trend over blocks (average of 10 stimuli)	Overall decrease in amplitude, more pronounced for the HAM genotype
Eitner et al., 2018*	N = 29	Short term	Electrical	Dorsum of the dominant hand	100	Train of 3 200 µs stimuli with 5 ms between pulses	4-6 seconds	NRS: after each block EEG: N1P1 and P0N1 amplitude	Percentage decrease between 2 and 14 minutes of stimulation	Decrease in pain ratings and N1P1 amplitude, not in P0N1 amplitude. Stronger reduction with conditioned pain modulation than with habituation protocol
Greffrath et al., 2007*	N = 16	Short term	Heat (thermode)	Volar side of the right forearm	30	Rising to peak and immediately returning to baseline	8-10 seconds	NRS: single trial EEG: N2-P2 amplitude	Percentage decrease and fit of exponential decay	Fast initial decrease for pain ratings and evoked potentials when stimulating at the same location, slow steady decrease for variable location
Hüllemann et al., 2013*	N = 13	Short term	Heat (laser)	Dorsum of the right hand (before and after testing one block at left hand)	100	5 ms	8.4-15.6 seconds	VAS: single trial EEG: N1 and N2-P2 amplitude	Trend over blocks	Decrease in pain ratings over blocks No effect for N1. Decrease of N2-P2 over blocks
Hüllemann et al., 2015*	N = 10 (control), N = 13 or N = 14 for capsaicin condition	Short term	Heat (laser)	Dorsum of the right hand	100		8.4-15.6 seconds	NRS: single trial EEG: N2-P2 amplitude	Trend over time	Decrease in pain ratings for control and capsaicin groups. Diminished N2-P2 habituation for stimulation in the primary area (after capsaicin) vs secondary area and controls
Hüllemann et al., 2016*	N = 12	Short term	Heat (laser)	Dorsum of the right hand	100	5 ms	8.4-15.6 seconds	VAS: single trial EEG: N1 and N2-P2 amplitude	Trend over blocks	Decrease in pain ratings and N2-P2 amplitude over blocks
Kersebaum et al., 2021*	Young = 34 (mean age = 24.9 years), older = 23 (mean age = 60.6 years)	Short term	Heat (laser)	Dorsum of the right hand	100	5 ms	8-12 seconds	NRS: single trial EEG: N2-P2 amplitude	Trend over time and high temporal resolution analysis	Decrease in N2-P2 habituation with age
Mancini et al., 2018	N = 16	Short term	Heat (laser)	Dorsum of the left or right hand	60	4 ms	1 second	EEG: N1, N2-P2, N4	Singular value decomposition	Characterization of the ERPs, the N1 and N2-P2 have similar decay functions and the habituated response has a similar waveform as nonhabituated response
Matre et al., 2015*	N = 21	Short term	Electrical	Volar forearm	90	11 ms (2 0.5 ms pulses separated by 10 ms)	10-15 seconds	VRS: single trial EEG: N2-P2 amplitude	Multilevel model with average per block	Decrease over blocks for ratings and N2-P2 amplitude, no interaction with sleep
Ødegard et al., 2015*	N = 33	Short term	Heat (laser)	Right dorsal hand	40	—	6-10 seconds	NRS: single trial EEG: N2-P2 amplitude	Trend over blocks	Decrease over blocks for N2-P2 amplitude, no interaction with sleep
Pazzaglia et al., 2016*	N=9 (verbal session) N=9 (conditioning session)	Short term	Heat (laser)	Dorsum of the left and right hand	25	—	10 seconds	VAS: after session EEG: N1 and N2-P2 amplitude	% change compared with baseline	Decreased habituation for treated hand (nocebo) compared with untreated hand for ratings and N2-P2 amplitude
Ruscheweyh et al., 2013*	N = 25	Short term	Heat (thermode)	Left and right volar forearm	20	Rising to peak and immediately returning to baseline	12-15 seconds	VAS: single trial CHEP: N2-P2 amplitude	Trend over blocks	Similar habituation effects at first session and second session (6 months apart) for both ratings and N2-P2 amplitude
Santoro et al., 2020*	N = 11	Short term	Heat (laser)	Dorsum of the left hand and left perioral region of the face	75	—	8-12 seconds	VAS: average per block EEG: N2-P2 amplitude	Trend over time Trend over time	No decrease in pain ratings Decrease of the N2-P2 amplitude for the third compared with first block for face stimulation
Schuh-Hofer et al., 2014	N = 12	Short term	Heat (laser)	Dorsum of the left and right hand	20	1 ms	6-9s	EEG: N1, N2, P2 amplitude	Percentage decrease in second compared with first part	Increased habituation in the P2 component after sleep deprivation, specifically under focusing and neutral conditions (as opposed to a distraction condition)
Vossen, van Breukelen, van Os et al., 2011*	N = 85	Short term	Electrical	Left middle finger	150	10 ms	9-11 seconds	NRS: single trial EEG: P1, N2, and P3 amplitude	Multilevel model	Correlation of ratings with amplitude (various directions)
Vossen, van Breukelen, Hermens et al., 2011 (same data)*	N = 85	Short term	Electrical	Left middle finger	150	10 ms	9-11 seconds	NRS: single trial EEG: N1, P1, N2, P2 amplitude	Trend over blocks and multilevel model	Overall comparable effects between habituation analyses but multilevel model has several benefits
Vossen et al., 2013 (same data)*	N = 76	Short term	Electrical	Left middle finger	150	10 ms	9-11 seconds	NRS: single trial EEG: ERFIAs (20 ms epochs)	Multilevel models	Habituation effects seen from 100 to 160 ms and 180 to 580 ms indicating a smaller ERFIA with higher trial number

* Studies that included self-report. The number of stimuli is reported for the habituation measurement and not necessarily referring to the total number of stimuli used in the study. If participants received 2 conditions with each 10 stimuli and habituation is calculated per condition, 10 stimuli are reported for the habituation analysis.

#### 3.2.2. Population characteristics

Age and sex have been investigated with respect to population characteristics (see Tables 2 and 3). With age differences, habituation trajectories were shown to slightly differ over time, as in particular, older subjects showed reduced habituation and in general more pain.^21^ Conversely, no effect of age was shown in a recent study.^49^ Studies focusing on sex differences reported mixed results. Three studies reported no sex differences in habituation with the use of either a short-term^32,97^ or a long-term paradigm.^11^ Conversely, 2 studies reported increased habituation in women as compared with men, which might have been related to use of lower stimulation intensities for women.^18,38^ When using the same stimulus intensity, habituation was present for both sexes, and no differences in habituation strength were shown.^39^ Jepma et al.^47^ also reported habituation to be present in both sexes, although it was more pronounced in women. Overall, sex effects for habituation to pain do occur but likely depend on the use of a specific paradigm, the calibration of stimuli, and/or used stimulation intensity.

**Table 4:** Habituation to pain: fMRI studies.

	Sample size	Timescale	Type of stimuli	Site	Nr of stimuli for habituation analysis	Duration	ISI	Habituation measurement	Habituation analysis	Main habituation result
Bauch et al., 2017	N = 20	Short term	Electrical	Dorsum of the right hand	40	500 ms	3 seconds	fMRI: BOLD	Whole-brain contrast	Activity decreased in the placebo group over blocks in the left postcentral gyrus and left MCC, but not for the haloperidol group
Becerra et al., 1999	N = 6	Short term	Heat (thermode)	Dorsum of the left hand	4	29 seconds	36 seconds	fMRI: BOLD	ROI activity compared between stimulus 1+2 vs 3+4	Decrease in the frontal gyrus, insula, cingulate gyrus
Bingel et al., 2007/2008*	N = 16/N = 10	Long term (8 days) + day 22 and 1 year later	Heat (thermode)	Left volar forearm	480	6 seconds	—	fMRI: BOLD VAS: average over 6 stimuli	ROI activity compared between day 1 and day 8 Trends over time	Decreased activity on day 8 compared with day 1 in the medial thalamus, bilateral anterior insula, contralateral S2, and bilateral putamen. Increased activity in the rACC. Pain ratings decreased over days After one year both ratings and activity were similar to day 1
Ellerbrock et al., 2015*	N = 40 (nocebo context n = 20 and control group n = 20)	Long term (21 days)	Heat (thermode)	Left volar forearm	1260	6 seconds	5 seconds	fMRI: BOLD VAS: average over 6 stimuli	ROI activity compared between days Multilevel models	Overall decrease in activity in bilateral anterior insula and bilateral S2. Increase in activity in the rACC. Nocebo context group showed increased activity in the opercular cortex. Generally decrease of pain ratings over days, increase within days. Less pronounced habituation and more pronounced sensitization in the nocebo context group. In the control group, intensity ratings parametrically varied with activity in the insula and PAG
Hahn et al., 2013	N = 22	Short term	Electrical	Dorsum of the left hand	20	500 ms	8-12, anticipation cue 5-15 seconds	fMRI: BOLD	Whole-brain contrast of run 1 vs run 4	3T: Decreased activity in the MCC, S2 and ventral anterior part of the insula in run 4 compared with 1 7T: Decreased activity in the dorsal posterior insula, PAG, and supramarginal gyrus
Ibinson et al., 2004	N=6	Short term	Electrical	Between right wrist and forearm	8	30 seconds	30 seconds	fMRI: BOLD	Whole-brain run 1 vs run 2 Signal change in ROIs	No significant effect No effect over runs Significant effect over stimuli (within run) in ACC and S1
Loggia et al., 2013	Met/Met = 12, Val/Met = 22, Val/Val = 20	Short term	Heat (thermode)	Right volar forearm	8	12 seconds	24-30 seconds	fMRI: BOLD	Whole-brain contrast	Met/Met genotype showed increased activity in second run in the PAG, hippocampal formation, lingual gyrus, calcarine cortex, precuneus, cuneus, superior and middle occipital gyri, and cerebellum
Lutz et al., 2013*	Meditation n = 14 and novices group n = 14	Short term	Heat (thermode)	Left forearm	16	10 seconds	93.3 seconds	fMRI: BOLD VAS: single trial	Trend over time in ROIs Trend over time	Expert meditators showed greater reduction across blocks in posterior insula, midinsula, and S2 No significant decrease over time for pain ratings
Mobascher et al., 2009	N = 12	Short term	Heat (laser)	Dorsum of the left hand	60	1 ms	8-12 seconds	fMRI: BOLD	Whole-brain contrast of first vs second half of experiment	No cluster-corrected activation
Mobascher et al., 2010	N = 32	Short term	Heat (laser)	Dorsum of the left hand	60	1 ms	8-12 seconds	fMRI: BOLD	Whole-brain contrast of first vs second half of experiment	More activity in first half in contralateral S1, bilateral S2, insula, and posterior ACC/MCC. No significant results in reverse contrast
Paul et al., 2021*	N = 23	Short term	Electrical	Dorsum of the left hand	20	500 ms	8-12, anticipation cue 5-15 seconds	fMRI: BOLD VAS: single trial	Whole-brain contrast of run 1 vs 4 Linear regression in ROIs Trend over time	More activity in first run compared with fourth run in bilateral inferior frontal gyrus, insula, bilateral S1 and S2, aMCC Linear decrease over runs in bilateral IFG, bilateral S2, and aMCC Linear decrease within runs in bilateral IFG and aMCC No decrease in ratings both across and within runs
Rodriguez-Raecke et al., 2010*	N = 38 (context = 18 and control group = 20)	Long term (8 days + day 90)	Heat (thermode)	Left volar forearm	480	6 seconds	—	fMRI: BOLD VAS: average over 6 stimuli	Whole-brain contrast between days Trends over time	Increased activity of the right operculum in the context group compared with the control group (uncorrected) Overall decrease in pain ratings over time—the context group remained stable, whereas the control group habituated

* Studies that included self-report. The number of stimuli is reported for the habituation measurement and not necessarily referring to the total number of stimuli used in the study. If participants received 2 conditions with each 10 stimuli and habituation is calculated per condition, 10 stimuli are reported for the habituation analysis.

ACC, anterior 1cingulate cortex; BOLD, blood-oxygen-level-dependent; IFG, inferior frontal gyrus; MCC, midcingulate cortex; PAG, periaqueductal gray; S1, primary somatosensory cortex; S2, secondary somatosensory cortex.

#### 3.2.3. Expectations and attention

Several psychological variables such as expectations and attention were reported to influence habituation (see Tables 2 and 4). In a study about expectations, participants were instructed that they would either habituate or sensitize over time, and this resulted in decreased or stable responses, respectively.^20^ Furthermore, a nocebo manipulation resulted again in stable, that is, not habituated, pain ratings over several days, whereas controls not exposed to this manipulation habituated as usual.^78^ A similar study showed long-term-habituation in both a control and a nocebo context groups over 21 days, although effects in the nocebo context group were less pronounced.^25^ Interestingly, when rating imagined heat pain, participants reported sensitization, whereas the actual rating of physical stimuli led to habituation.^27^ An unexpected change in electrical intensity was also reported to affect habituation.^3,4^

Attention and fear have also been linked to habituation for modulating expectations and information processing. Yet, results are still diverse, and large-scale investigations are lacking. For instance, when attention was modulated, distraction resulted in habituation, whereas attention resulted in the absence of habituation.^2^ Anxiety did not affect habituation in the same study.^2^ Furthermore, reduced habituation to pain has been shown in participants with higher levels of pain-related catastrophizing^22^ but not in participants with sensory overresponsiveness.^107^

Differences between sensory and affective aspects of habituation were investigated in 2 long-term heat pain studies showing both habituation of pain intensity and pain unpleasantness over days.^11,28^ Breimhorst et al.^11^ reported an increase in both pain and unpleasantness within days, whereas, interestingly, the slope within these days diminished across days. Conversely, a preliminary short-term study did not report a decrease in pain and unpleasantness.^58^

In conclusion, expectations and attention are shown to modulate habituation to pain on a short-term and long-term timescale. Both sensory and affective aspects of pain are subject to habituation, although, sometimes, conflicting evidence is presented in the literature with respect to effects of anxiety and attention.

#### 3.2.4. Experimental settings

Studies with respect to experimental settings showed that habituation to pain depends on spatial summation (ie, small distance between probes reduced habituation) and (initial) stimulation intensity (stronger habituation for lower (initial) stimulus intensities).^18,39,47^ This latter point is in line with the properties listed by Rankin et al.^75^ but is not always observed. In some studies, the rate of habituation appears to be the same, but only the amplitude (ie, level of the rating) increases with higher stimulus intensities, for example ^28,109^. The site of stimulation might also play a role. For instance, stimulation on the forehead resulted in larger within-session sensitization than stimulation on the arm.^86^ Habituation over days did not show any differences with respect to the stimulation site. Site-specific effects were further investigated in a paradigm with 3 runs of heat stimulation, where within each run a different (adjacent) site was stimulated, which was repeated in the next runs.^47^ This study showed sensitization within the first run and habituation across the 3 runs, potentially reflecting site-nonspecific sensitization and site-specific habituation, respectively.^47^ Greffrath et al.^32^ also reported a strong initial habituation effect for stimulation on the same site and a steady, but slow, decrease when using a variable stimulation site. A central component of habituation to pain was subsequently tested by investigating transfer effects to the nonstimulated arm. In this paradigm, participants are stimulated several times on one arm (“stimulated arm”) and then once more at the other arm (“nonstimulated arm”), thereby investigating whether the participant would report a similar or different intensity. Although stimulation at the nonstimulated arm resulted in an increase of pain (ie, no continued habituation or transfer effect) in one study,^28^ another study showed habituation to pain for both participant's stimulated and nonstimulated arms.^76^ This suggests involvement of both spinal and supraspinal mechanisms.^76^ Therefore, the intensity and site of stimulation seem to affect habituation to pain, in particular with the use of repeated heat stimulation on the same site.

#### 3.2.5. Investigating mechanisms of habituation

Treister et al. (2010) compared different modes of endogenous pain modulation, namely, habituation and diffuse noxious inhibitory control (DNIC, see Table 2). DNIC involves the inhibition of nociceptive input at the spinal level when (simultaneous) noxious stimulation is taking place elsewhere on the body. It was shown that both habituation and DNIC resulted in decreased pain intensity, with a significant correlation between these effects, indicating a general endogenous analgesic effect. In an offset analgesia paradigm, which is a different endogenous pain modulation paradigm, the stimulus temperature is increased for a few trials and then returned to baseline, which usually results in a disproportionately large reduction in pain perception. In this paradigm, habituation effects were shown in the higher temperature trials, indicating that several processes might occur simultaneously.^93^ Furthermore, the development of habituation was shown to be unaffected by the opioid receptor antagonist naloxone. The authors concluded from this that the underlying mechanism does not directly involve the endogenous opioid system.^76^ In summary, inhibitory modulation of pain, as for instance, DNIC or offset analgesia, may show similar decreases in pain as noted by habituation. Hence, the investigation of potential common pathways involved in either DNIC or habituation to pain would be an interesting direction for future research. The endogenous opioid system does not seem to be involved.

#### 3.2.6. Reliability of habituation effects over time

Use of different stimulation timescales may also effect habituation to pain. In a paradigm investigating effects over different timescales, it was shown that in this study, repetitive brief pulses (max 1.8 Hz) induced sensitization; however, over trials (within runs), this resulted in habituation to pain, and across runs, the ratings were stable.^51^ Therefore, it is important to distinguish and compare analyses over different timescales because they might show opposing effects. Analyses of intersession variability for pain intensity also revealed decreased habituation to pain over sessions, for different temperatures.^72^ Nevertheless, an earlier study with a similar design, but smaller sample, did not show any habituation effects over sessions.^81^ Furthermore, a recent study showed that pain ratings decreased within a block and remained stable over blocks.^16^ However, a comparison of pain ratings between 2 visits showed very low reliability.^16^ Overall, reliability of estimates of habituation to pain across timescales seems rather limited, and the extent to which habituation occurs across sessions and timescales needs to be determined.

#### 3.2.7. Individual differences

Importantly, large interindividual variability has been shown in rating data, ranging from sensitization to habituation to pain within a run of heat stimuli.^47^ These individual differences were also noticed in a long-term heat study, where no habituation effects over time across the group of healthy volunteers (n = 27) were shown as compared to controls without any stimulation.^92^ Nevertheless, behavioral data demonstrated that a subgroup of participants (n = 14) sensitized, whereas the others (n = 13) habituated over the stimulation days. Similar long-term paradigms observed habituation on the group level but sensitization in 25% of the participants.^9,77^ Individual differences have also been noted in a study by Slepian et al.^88^: 25% of participants reported habituation, but the group-level analysis showed a sensitization effect.^88^ These individual effects are thus very important to report and must be taken into account for a comprehensive understanding of habituation to pain.

#### 3.2.8. Conclusions

Self-report studies have shown that psychological aspects, such as expectations, are able to influence habituation to pain. The effects of population characteristic on habituation, like age and sex, are not yet conclusive. Experimental settings, such as the number of stimuli and stimulation site, also influence habituation to pain and results vary depending on the investigated timescale. Habituation to pain might have common mechanisms with other pain modulation paradigms. Individual differences should be the focus of future research because this will provide detailed insight into the variability in habituation to pain.

### 3.3. Habituation to pain: electroencephalography

#### 3.3.1. Type of stimuli

In contrast to self-report studies, which mainly used heat stimulation with a thermode (section 3.2.1), most EEG studies used heat stimulation using a laser and electrical stimulation (Fig. 3). Brief stimulation allows measuring evoked potentials, such as the N1, P1, N2, or P2 amplitude. Contact heat–evoked potentials (CHEPs) have been developed over recent years and consist of very brief heat stimuli with reduced rising times compared with standard thermode heat stimuli, which make them more compatible with EEG.^31^ In this review, we adhere to the description of EEG components as used in the original articles (eg, N2-P2 amplitude, when it is described as such in the article).

#### 3.3.2. Population characteristics

In view of habituation to pain based on EEG recordings, population characteristics, such as age and genetic differences, have been studied (Table 3). Habituation of the N2-P2 amplitude was shown to be affected by a genotype involved in monoaminergic activity and regulators of gene expression.^19,84^ Age effects on the N2-P2 amplitude showed an increase in habituation over several groups between 18 and 72, although there were no differences between the groups after a Bonferroni correction.^17^ Another study reported a decrease in the N2-P2 amplitude in participants between 50 and 70 compared with young subjects (mean age = 24.9 years).^49^ Effects of sleep (or the lack thereof) have not been found to affect habituation for both pain ratings and the N2-P2 amplitude. This was observed regardless of whether a crossover within-subject design^61^ or a randomized between-subjects design^66^ was employed. Conversely, in another crossover within-subject design, Schuh-Hofer et al.^87^ observed increased habituation of the P2 component after sleep deprivation, in both a neutral and focusing condition, but not during distraction. However, habituation was not related to individual sleepiness scores, and the sample was considerably smaller than the first 2 studies.^87^ In conclusion, sleep and age do not seem to affect habituation to pain in a consistent way. Furthermore, there are indications that several genotypes may affect habituation to pain. These population characteristics should be considered in future studies.

#### 3.3.3. Expectations and attention

EEG research indicates that the expectation to feel more pain decreases habituation of the N2-P2 but not the N1 amplitude (Table 3),^69^ although this has not been replicated in larger-scale studies. Habituation of the N1 and P2 component was also shown when investigating different attention and distraction tasks (such as mental arithmetic), indicating that attention or distraction did not affect habituation to pain.^10^

#### 3.3.4. Experimental settings

Paradigms using laser-evoked potentials (LEPs) have consistently shown a decrease in N2-P2 amplitude over blocks of stimuli (Table 3).^41,42^ In addition, LEP-evoked habituation to pain was investigated under the influence of capsaicin-induced peripheral and central sensitization.^43^ Capsaicin-induced central sensitization did not alter the LEP-evoked habituation, whereas at the same time, habituation was shown to be reduced in the primary stimulation area after applying capsaicin.^43^ When using a fixed location for CHEPs, a fast decay of the N2-P2 amplitude followed by a plateau is reported.^32^ The stimuli at the fixed location may have resulted in fatigue at the sensory afferents, that is, peripheral habituation. With the use of a variable location, a gradual decrease in N2-P2 was observed, and therefore, central habituation is likely to occur.^32^ In conclusion, EEG recordings and experimental settings can be used to determine and discriminate between peripheral and central sensitization as well as peripheral and central habituation to pain.

#### 3.3.5. Investigating mechanisms of habituation

In research on conditioned pain modulation (CPM), a study compared habituation and CPM effects^24^: here, the CPM showed a stronger reduction in EEG amplitude as compared to the habituation protocol. It was concluded that the underlying mechanisms of CPM and habituation might partially overlap.^24^

#### 3.3.6. Reliability of habituation effects over time

A reproducibility study using CHEPs reported similar habituation to pain over 2 sessions (6 months interval), although the average amplitude was increased during the second session.^83^ A recent study reported no effects on habituation to pain within and across blocks for the N2-P2 amplitude, and starting values (first trials) were similar between the 2 visits.^16^

#### 3.3.7. Analysis methods and statistical approach

Use and implementation of state-of-the-art analysis methods and/or correct statistical approaches are of utmost importance for correct and optimal interpretation of results. The current standard is looking at trends over time in evoked potentials, where effects are not always established (Table 3).^12^ The use of new analysis methods demonstrated the presence of habituation to pain on a finer timescale.^104^ Multilevel modelling showed the benefit of using single trial data and including decay functions such as quadratic effects to model habituation over time. Moreover, it is possible to take into account individual differences by including a random intercept and/or slope in the model. This statistical approach of multilevel modelling was extended by the event-related, fixed-interval area (ERFIA) technique, which partitions the data into small epochs, calculates the area under the curve, and uses this as dependent variable in a multilevel model. This enabled demonstration of habituation effects outside the peak amplitude, for example, between 100 ms and 160 ms after stimulus onset.^103^ A higher temporal resolution was also pursued in a paradigm with LEPs, to minimize the required stimuli.^49^ Another approach in characterizing the event-related potential (ERP) waves is the use of singular value decomposition.^59^ This approach revealed similar decay functions of N1 and N2-P2 waves and similar waveforms for habituated and nonhabituated trials.^59^ New EEG analysis methods and modeling combined with advanced statistical approaches allows improved and detailed analysis of habituation to pain.

#### 3.3.8. Conclusions

EEG studies have significantly contributed to our understanding of habituation to pain. Habituation to pain seems to play an important role in the reduction of the N2-P2 amplitude, although this is less robust for the N1 amplitude. Although effects of genotype, sleep, and distraction are still inconclusive, different experimental settings allow to investigate both peripheral and central effects of habituation to pain. New analysis methods and state-of-the-art statistical approaches further allow for a more profound understanding of mechanisms underlying habituation to pain.

### 3.4. Habituation to pain: functional magnetic resonance imaging

#### 3.4.1. Changes in brain activity on different timescales

Pain is associated with activity in several brain areas including the somatosensory cortices, insula, cingulate cortex, periaqueductal gray, and prefrontal cortex.^60,110^ A decrease in activity across (sequences of) pain stimuli has been interpreted as habituation, although this still is a matter of discussion (see also Section 4.1 Challenges in the field). In neuroimaging fields outside pain, this activation decrease has also been described as the repetition suppression effect, but the differences with habituation have not been systematically reviewed.^5^ Short-term habituation to pain with the use of fMRI has been investigated using analyses over different time windows (Table 4).^7,36,45,63,64,68^ Over the course of 4 heat stimuli, brain activity decreased in several areas, including the bilateral frontal gyrus, left insula, and bilateral anterior cingulate gyrus.^7^ A similar study using 2 runs of 4 electrical stimuli showed a decay in activity in the left anterior cingulate cortex (ACC) and left primary somatosensory cortex (S1) over the 4 stimuli but not over the 2 runs.^45^ Two subsequent studies used heat stimuli with a laser and contrasted stimuli of the first half with the second half of the experiment.^63,64^ Although the first study did not show any habituation, the second (more powered) study showed increased activity in the first half of the experiment in the contralateral S1, bilateral secondary somatosensory cortex (S2), bilateral insula, the posterior ACC/midcingulate cortex (MCC), bilateral supramarginal gyrus, and bilateral parietal operculum (see Supplemental Table 1 for a complete overview, available at http://links.lww.com/PAIN/B920).^63,64^ Furthermore, a study comparing several runs of electrical stimulation reported decreased activity in the MCC, left S2, and ventral anterior part of the right insula.^36^ Similarly, a recent work reported that the bilateral inferior frontal gyrus (IFG), including anterior parts of the insula, bilateral S1/S2, and the anterior MCC (aMCC), showed decreased activity over runs.^68^ Moreover, activity in the aMCC was specific to habituation of painful compared with nonpainful stimuli, thereby indicating a role in pain identification.^68^ In addition, exploratory analyses also indicated habituation within runs in the IFG and aMCC. The groundwork for fMRI long-term habituation to pain studies was conducted by Bingel et al.^9^ with a study involving 8 daily stimulation sessions. Repeated painful heat stimulation resulted in long-term habituation because changes in brain activity were observed on day 8 compared with day 1, which then remained stable until day 22. Over the 8 days, brain activity decreased in the bilateral anterior insula, the medial thalamus, contralateral S2, and bilateral putamen, whereas increased activity over time was found in the rostral ACC (rACC), specifically the subgenual ACC (sgACC).^9^ Therefore, the sgACC was suggested to be involved in antinociceptive activity and mediation of (long-term) habituation to pain. The brain activity in the sgACC was shown to return to baseline (ie, similar to day 1) in a 1-year follow-up study.^8^

**Figure 4.**
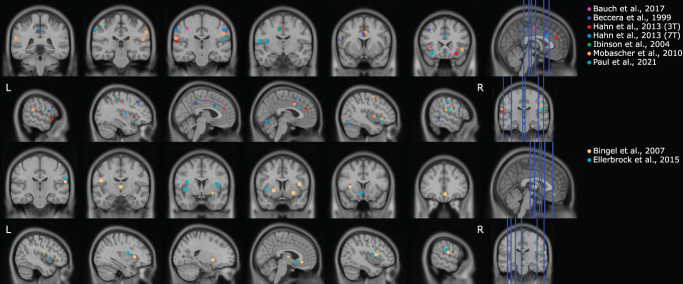
Overview of fMRI studies involving habituation to pain projected on an MNI brain. Centers of peak activity are visualized as 5-mm spheres. Only data corrected for multiple comparisons are included, and we did not include between-group analyses for factors of influence. For short-term habituation (upper 2 rows), spheres represent decreasing activity over time. Several timescales are included, which can be found in Table 9 and Supplementary Table (available at http://links.lww.com/PAIN/B920). For long-term habituation (lower 2 rows), spheres indicate areas in which activity decreased over time, except for the rACC/sgACC, which showed increased activity (column 4, row 4).

In conclusion, fMRI studies demonstrated neural habituation effects for sequences as short as 4 stimuli, ranging to long-term effects over several runs, mostly located in the cingulate cortex, insula and somatosensory cortices (see Supplementary Table 1, available at http://links.lww.com/PAIN/B920). The S2 and insula were sensitive to both short-term and long-term habituation, whereas activity in the ACC/MCC seems to be unique for short-term habituation. By contrast, activity in the sgACC/rACC seems to be related to long-term habituation only (Fig. 4).

#### 3.4.2. Expectations and mechanisms of habituation

Specific genotypes,^56^ the practicing of meditation,^57^ or administration of haloperidol (a D2-dopamine receptor antagonist)^6^ have all been suggested to affect brain activity during repeated painful stimulation. The effect of haloperidol is of specific interest because it suggests that especially the dopaminergic network is involved in the process of short-term habituation.^6^ The results of this randomized, double-blind, within-subject fMRI study showed that, only when participants received the placebo, they habituated (over 2 runs of 3 different stimulus intensities), as indicated by decreased activity in the left postcentral gyrus and left MCC. This habituation effect was not found when those participants received haloperidol. In fMRI studies investigating the effect of a nocebo manipulation, the nocebo group showed increased brain activity over time in the right operculum (although uncorrected for multiple comparisons).^78^ Similarly, another study reported increased activity over time in the opercular cortex for the nocebo group.^25^ In conclusion, a nocebo manipulation has shown to affect the habituation process both on a self-report and on a neural level. The dopaminergic network is suggested to underlie habituation to pain because of the habituation-blocking effect of a dopamine antagonist.

### 3.5. Habituation to pain: association between self-report and electroencephalography/functional magnetic resonance imaging

Although about half of the EEG and fMRI studies also included pain ratings (with sometimes opposing effects between neural and self-reported measures), only a limited number of studies directly tested this association. For fMRI, Ellerbrock et al.^25^ reported that ratings were positively related to activity in the insula and periaqueductal gray (Table 4). For EEG, Greffrath et al.^32^ reported that pain ratings and the N2-P2 amplitude were positively related, but Matre et al.^61^ found no relation between the 2 measures (Table 3). A positive relation was reported between a decrease in pain ratings and decrease in the N2-P2 amplitude, thereby specifically testing the habituation to pain effect.^41^ EEG using multilevel modelling reported both positive and negative associations between pain ratings and EEG amplitude.^105^ Moreover, this association was found to decrease with a higher trial number, that is, habituation affects the association.^105^ The preliminary results on this topic thus suggest an association between neural habituation (as measured with EEG) and self-report ratings. However, it should be noted that most studies did not measure behavioral and neural habituation concurrently or showed opposing (but not statistically tested) effects. Clearly, this issue needs further in-depth investigation and remains an important topic for follow-up studies.

## 4. Discussion

In this scoping review, we included 63 studies on habituation to pain through measures of self-report or brain activation (EEG and/or fMRI). We found a large variety in methods, experimental settings, and contexts, suggesting that habituation is a ubiquitous phenomenon. Self-report studies (section 3.2) have shown a large influence of expectations and stimulation site, as well as the presence of individual differences in habituation to pain in healthy individuals. EEG (section 3.3) and fMRI (section 3.4) studies have shown characteristics of neural habituation to pain such as involvement of the N2-P2 amplitude and several identified brain areas (cingulate cortex and somatosensory cortices). Furthermore, the measured timescale showed different effects of habituation (eg, within run or across), and it is important to further develop and use statistical methodology to improve analyses and interpretations of these effects.

The majority of studies have used heat stimuli with a thermode, followed by heat using a laser and electrical stimuli (Fig. 3). Although the effects of heat stimuli with a thermode as compared with heat stimuli using a laser and electrical stimulation have not been systematically compared, all types of stimulation have shown to result in habituation to pain. Peripheral effects might occur more often when using thermode or laser heat stimuli on the same site, with short intervals or with intraepidermal electrical stimulation.^32,47,65^ In one such study, it was shown that both the N2-P2 amplitude and pain ratings decreased, suggesting that these peripheral effects are also reflected in brain activity.^32^ Furthermore, habituation effects shown after changing the stimulation site suggest the involvement of central processes in habituation. However, results from self-report studies were inconclusive.^28,76^ This inconsistency suggests that peripheral and central effects related to habituation are likely intertwined. The underlying mechanisms of habituation to pain are still under investigation. Although at the micro-level habituation has been suggested to involve a decrease in cell excitability or homosynaptic depression of excitatory neurotransmission,^62^ it is still unclear how this translates to the macro-scale effects. Besides, habituation studies often show similar outcomes as compared with other endogenous pain modulations, such as CPM and/or DNIC^24,97^ or conditioning using low-frequency stimulation.^35,50^ Furthermore, there is preliminary evidence for the involvement of the dopamine system.^6^ In addition, several specific cortical areas were reported to be involved in habituation to pain, particularly the cingulate cortex, insula, and somatosensory cortices.^68^ The EEG studies mostly reported effects on the N2-P2 amplitude. For LEPs, this N2-P2 effect has been hypothesized to originate from the cingulate cortex and S2.^29,100^

### 4.1. Challenges in the field

This review highlights the diversity of approaches and variations in experimental settings. The sample sizes used in the various studies were relatively small, which, in combination with the variations in methodology, makes interpretation and the formulation of general conclusions difficult. As a result, the evidence related to the degree and/or presence of habituation to pain cannot be regarded as very robust. For example, population characteristics such as age and sex showed mixed results in self-report (3.2.2) and EEG (3.3.2) measures. Conversely, expectations have shown robust effects on self-report ratings (3.2.3), either increasing or decreasing habituation effects. The latter indicates that the process of habituation is inherently subject to experimental settings such as the context and the given instructions, which should be taken into account when comparing studies. Future work could improve standardization across participants in terms of task context and expectations about habituation or sensitization.

Furthermore, terminology is not always consistent. For example, “adaptation,” “decay,” and “habituation” have been interchangeably used in the literature to describe highly similar response decrements. Moreover, in the field of neuroimaging, “repetition suppression” has been used to indicate diminished neural activation.^5^ Therefore, it is important to have a uniform use of terminology. Similarly, distinctions between levels of habituation and/or sensitization should be clearly indicated. For example, when “diminished habituation” is reported, this could also indicate sensitization to pain (ie, when there is an increase with respect to the first stimulus) and should be described as such.

Another point of terminology is the concept of dishabituation, which is closely related yet distinct from habituation. Dishabituation is defined as “presentation of a different stimulus that results in an increase of the decremented response to the original stimulus” (Rankin et al., 2009, p. 137).^75^ Dishabituation should not be confused with sensitization because dishabituation indicates an increase in a previously habituated response and not merely an increase. Although outside the scope of this review, it is important to note that several studies have investigated dishabituation. For example, within repeated triplets of stimuli with 1 second of interstimulus interval, a decrease was shown in the N2 and P2 peak, but changing the modality or increasing the stimulus intensity of the third pulse induced an increase in amplitude (ie, dishabituation); however, a change in spatial location did not affect the amplitude.^44,79,96,99^ There are also studies using other paradigms that discuss dishabituation. For example, Scheuren et al.^85^ included an intervention to induce secondary hyperalgesia, and this has shown to result in dishabituation of the original evoked potential. Thus, various paradigms have been developed to increase our understanding of dishabituation.

Results and paradigms are also often named after the intended effect (eg, habituation paradigm). However, it is likely that there is no specific paradigm to test for either habituation or sensitization because these processes largely overlap (with the exception of temporal summation, although even there, habituation effects have also been shown; see Edwards and Fillingim^21^). The results of this review indeed show that the few studies that report individual differences include a large variability over time with sensitization instead of habituation effects in some participants. This might be in line with the dual process theory (see introduction Section 1.0), stating that habituation and sensitization occur independently but may interact and then result in a behavioral response.^34^ Furthermore, a low stimulus intensity has been shown to result more often in habituation as compared with sensitization.^18,47^ This phenomenon may result from the lower threat it poses to the body.^15^ However, in some cases, the rate of habituation is similar for different intensities, and only the amplitude is increased for higher stimulus intensities (ie, level of the rating).^28,109^ Therefore, it remains unclear whether habituation and sensitization are a person-specific “trait” and stable under the same experimental settings or whether the influence of expectations, mood, and social context results in a “state” effect.

The inclusion of ratings as a measure of habituation to pain remains a topic of debate in the field. Including ratings after each trial might increase attention to the pain stimuli; however, including ratings only at the end of a block or experiment might lead to biased ratings through the habituation effects itself (eg, habituation occurred, and because most recent information is recalled (“recency effect”), lower ratings are reported).

**Figure 5.**
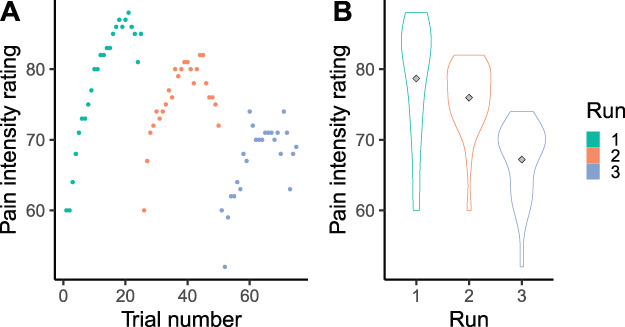
In panel A, individual pain intensity ratings are visualized that show an increase over trials within each run (sensitization). In panel B, the averages (grey diamond) of the same trials per run are visualized, which instead show a decreasing trend across runs (habituation).

The definition of habituation states “a decrease in response after repeated stimulation” but does not require any minimal or maximum number of repetitions. This results in a great variety in the number of repetitions being used in the field (see also Fig. 2), and it can be questioned whether 2 repetitions are sufficient to describe a habituation effect. Habituation effects also differed across timescales. Thus, in the case of averaging trials, this might mask effects on shorter timescales. Figure 5 shows an example, which indicates individual data where there is an increase of the pain intensity ratings over trials within a run, but when averaged, only a decrease across runs is visible. As for now, ratings per trial are encouraged because they provide more insight into the trajectories of habituation and sensitization to pain. Furthermore, multilevel models support including all trials and random effects for intercepts and slopes, thereby allowing for individual changes over time. Moreover, a multilevel model can accommodate effects both within and across runs. Therefore, this type of modeling is encouraged when analyzing effects over time.

From our review, we conclude that it has been challenging to align self-report ratings with neural habituation. Conceptually, it is unclear whether habituation to pain on the neural level (ie, a decrease in activity or amplitude) is sufficient to indicate habituation effects or whether self-report ratings are needed. A decrease in brain activity may be related to several underlying neural mechanisms, but these mechanisms not necessarily need to be related to habituation to pain. For example, Paul et al.^68^ demonstrated a decrease in fMRI BOLD activity in the cortical areas MCC, IFG, and S2 but reported no decrease in pain ratings. Interpreting the observed decrease in activity as habituation to pain^68^ thus remains a topic of further discussion. Moreover, changes in neural processes (eg, brain activity) may be better described as nociception instead of pain.^1^ Although most literature described habituation to pain, this remains an important challenge in the field with respect to EEG and fMRI research. Our review further shows that effects are often not aligned, in particular with respect to the direction of effects based on neural (EEG, fMRI) or self-report measures (see Section 3.5). Future studies will help to determine whether there are similar processes underlying self-report and neural habituation or that these 2 levels are separate forms of habituation to pain. Christoffersen^13^ already stated very early on that “at the least, it may serve the purpose of avoiding conceptual confusion if habituation at the 2 levels were named separately until a causative link between these levels has been proven to exist in each case” (pp. 61-62).

### 4.2. Future recommendations

Based on the current state of the field, the gaps in the literature, and the challenges in the field, we propose the following recommendations for future research.

First of all, with respect to terminology, studies should clearly describe the measured effects. Care should be taken with comparison of different operationalizations of habituation, such as measuring the pain threshold, ratings, skin conductance, or neural measures. It is important to specifically describe which type of measurement a study used, without making a conclusion based on several measurements. For example, there might be habituation of the pain threshold (which involves a process of tolerance) but not of pain stimuli above the threshold. Therefore, we recommend clearly describing the specific effect, for example, habituation to pain intensity ratings.

Second, care should be taken when discussing and comparing different timescales. Habituation was reported to differ based on various timescales and should be described as such, for example, within and across run effects. Within-run effects are more subject to peripheral habituation, especially when the same location is stimulated and/or if a break between the runs is present. Across run effects might be more robust and indicate central processes. However, habituation is also known to become more pronounced over series (also called potentiation).^75^ In addition, studies using long-term timescales demand a different involvement of participants and show habituation effects resulting from longer stimulation periods.

Third, large individual differences exist with respect to habituation to pain. Thus, studies should focus more on individual characteristics, which may lead to increased personalized approaches and treatments. Moreover, individual differences will lead to more insight into the expected effect, for example, whether a certain paradigm elicits habituation in each participant or only in a subset of participants.

Fourth, the use of individual calibration of the pain stimulus is encouraged, especially with self-report or the combination of self-report with EEG or fMRI (to ensure a standard procedure across modalities). With calibration differences in skin parameters and pain receptors taken into account (ie, limiting peripheral effects), differences in experienced pain are reduced (ie, reducing floor or ceiling effects where pain is too high for some participants), and the intensity of the stimulus may affect the likelihood of habituation and sensitization. Although a recent large study did not report a relation between perceived pain intensity and brain activation,^40^ calibration to individual thresholds may be very important for habituation and sensitization processes and the study of their neural correlates.

Fifth, because habituation may occur under many circumstances and contexts, it is of importance that studies involving repeated painful stimulation, test for effects over time or repetition and include this as a covariate in the statistical model even if the investigation of habituation or sensitization is not the primary focus.

A sixth, more generic, recommendation concerns the use of larger participant groups with preregistration and sharing of analyses/code. The present review clearly shows that most studies are based on a relatively low sample size (Fig. 2B). Large sample sizes will help in generalization of effects and identifying individual differences, whereas preregistration will make the expectations about habituation and/or sensitization to pain more transparent and will increase confirmatory analysis approaches.^37^

### 4.3. Limitations of the review

In this review, we did not include studies using behavioral measures such as reflexes, habituation of the pain threshold, and habituation of nonpainful stimuli, although the literature using these measures is substantial. Moreover, we deviated from the preregistered protocol by not including the literature on habituation to pain in chronic pain patients. This was decided because there was abundant literature to describe self-report and neural measures of habituation first in healthy individuals, whereas studies on habituation to pain in chronic pain patients have formed the fundament of a separate review.^102^

Furthermore, studies where sensitization to pain was the main outcome were excluded. The present review clarifies that further investigation is needed to highlight the distinctions between habituation and sensitization to pain and the possible underlying individual characteristics.

Another limitation is that effects of habituation to pain are not always described uniformly (eg, habituation, adaptation, decay or simply decrease in pain), potentially leading to some missed articles. Furthermore, a few articles quantified a decrease in pain but did not describe (or define) it as habituation. We decided to not make interpretations beyond those of the original articles, which then may result in not including these articles. This also applies to the use of the term pain instead of nociception. In the case of EEG and fMRI studies, the term habituation to nociceptive stimuli might have been more applicable. However, because this issue is outside the scope of the current review, the descriptions as used by the authors of the original papers were used (ie, habituation to pain/painful stimuli). In addition, some studies might have examined habituation effects but did not find and/or report this in the article (ie, null effects).

### 4.4. Conclusions

The results from this scoping review show that habituation to pain is a fundamental process that occurs under a variety of circumstances and is expressed both with self-reported and neural measures. Habituation to pain is present on very short (few stimuli) to long (several days) timescales, and the context (eg, expectations) and experimental settings (eg, stimulus intensity, stimulation site) can influence the degree of habituation based on self-reported and/or neural (EEG, fMRI) measures.

Future well-designed studies should address individual differences, population characteristics, and the relation between neural and self-report measures. In addition, the discrepancy between habituation and sensitization should be further verified, accompanied by consistent and clear use of terminology.

As of now, the insights from this review on habituation to pain in healthy individuals can be used to optimize designs and methods for measuring habituation to pain, and this will aid our understanding of the underlying mechanism. Individualized approaches can be used for exploring pain modulation in chronic pain patients.

## Conflict of interest statement

The authors have no conflicts of interest to declare.

## Appendix A. Supplemental digital content

Supplemental digital content associated with this article can be found online at http://links.lww.com/PAIN/B920.

## References

[1] Apkarian AV. Definitions of nociception, pain, and chronic pain with implications regarding science and society. Neurosci Lett 2019;702:1–2. 30503918 10.1016/j.neulet.2018.11.039 PMC6520170

[2] Arntz A Dreessen L Merckelbach H. Attention, not anxiety, influences pain. Behav Res Ther 1991;29:41–50. 2012588 10.1016/s0005-7967(09)80006-5

[3] Arntz A Lousberg R. The effects of underestimated pain and their relationship to habituation. Behav Res Ther 1990;28:15–28. 2302146 10.1016/0005-7967(90)90051-j

[4] Arntz A Van den Hout MA. Generalizability of the match/mismatch model of fear. Behav Res Ther 1988;26:207–223. 3408456 10.1016/0005-7967(88)90002-2

[5] Barron HC Garvert MM Behrens TE. Repetition suppression: a means to index neural representations using BOLD? Philosophical Trans R Soc B 2016;371:20150355. 10.1098/rstb.2015.0355 PMC5003856 27574308

[6] Bauch EM Andreou C Rausch VH Bunzeck N. Neural habituation to painful stimuli is modulated by dopamine: evidence from a pharmacological fMRI study. Front Hum Neurosci 2017;11:630. 29311880 10.3389/fnhum.2017.00630 PMC5742644

[7] Becerra LR Breiter HC Stojanovic M Fishman S Edwards A Comite AR Gonzalez RG Borsook D. Human brain activation under controlled thermal stimulation and habituation to noxious heat: an fMRI study. Magn Reson Med 1999;41:1044–1057. 10332889 10.1002/(sici)1522-2594(199905)41:5<1044::aid-mrm25>3.0.co;2-m

[8] Bingel U Herken W Teutsch S May A. Habituation to painful stimulation involves the antinociceptive system--a 1-year follow-up of 10 participants. PAIN 2008;140:393–394. 18952372 10.1016/j.pain.2008.09.030

[9] Bingel U Schoell E Herken W Büchel C May A. Habituation to painful stimulation involves the antinociceptive system. PAIN 2007;131:21–30. 17258858 10.1016/j.pain.2006.12.005

[10] Blom JHG Wiering CH Van der Lubbe RHJ. Distraction reduces both early and late electrocutaneous stimulus evoked potentials. J Psychophysiol 2012;26:168–177.

[11] Breimhorst M Hondrich M Rebhorn C May A Birklein F. Sensory and sympathetic correlates of heat pain sensitization and habituation in men and women. Eur J Pain 2012;16:1281–1292. 22407985 10.1002/j.1532-2149.2012.00133.x

[12] Bromm B Scharein E. Response plasticity of pain evoked reactions in man. Physiol Behav 1982;28:109–116. 7079309 10.1016/0031-9384(82)90111-1

[13] Christoffersen G Habituation: events in the history of its characterization and linkage to synaptic depression. A new proposed kinetic criterion for its identification. Prog Neurobiol 1997;53:45–66. 9330423 10.1016/s0301-0082(97)00031-2

[14] Condes-Lara M Calvo JM Fernandez-Guardiola A. Habituation to bearable experimental pain elicited by tooth pulp electrical stimulation. PAIN 1981;11:185–200. 7322602 10.1016/0304-3959(81)90004-X

[15] De Paepe AL Williams ACC Crombez G. Habituation to pain: a motivational-ethological perspective. PAIN 2019;160:1693–1697. 31335639 10.1097/j.pain.0000000000001533

[16] De Schoenmacker I Leu C Curt A Hubli M. Pain-autonomic interaction is a reliable measure of pain habituation in healthy subjects. Eur J Pain 2022;26:1679–1690. 35671124 10.1002/ejp.1990 PMC9544564

[17] de Tommaso M Ricci K Montemurno A Vecchio E. Age-related changes in laser-evoked potentials following trigeminal and hand stimulation in healthy subjects. Eur J Pain 2017;21:1087–1097. 28207185 10.1002/ejp.1010

[18] Defrin R Pope G Davis KD. Interactions between spatial summation, 2-point discrimination and habituation of heat pain. Eur J Pain 2008;12:900–909. 18280188 10.1016/j.ejpain.2007.12.015

[19] Di Lorenzo C Daverio A Pasqualetti P Coppola G Giannoudas I Barone Y Grieco GS Niolu C Pascale E Santorelli FM Nicoletti F Pierelli F Siracusano A Seri S Di Lorenzo G. The upstream Variable Number Tandem Repeat polymorphism of the monoamine oxidase type A gene influences trigeminal pain-related evoked responses. Eur J Neurosci 2014;39:501–507. 24494688 10.1111/ejn.12458

[20] Doganci B Breimhorst M Hondrich M Rodriguez-Raecke R May A Birklein F. Expectations modulate long-term heat pain habituation. Eur J Pain 2011;15:384–388. 20951617 10.1016/j.ejpain.2010.09.003

[21] Edwards RR Fillingim RB. Effects of age on temporal summation and habituation of thermal pain: clinical relevance in healthy older and younger adults. J Pain 2001;2:307–317. 14622810 10.1054/jpai.2001.25525

[22] Edwards RR Smith MT Stonerock G Haythornthwaite JA. Pain-related catastrophizing in healthy women is associated with greater temporal summation of and reduced habituation to thermal pain. Clin J Pain 2006;22:730–737. 16988570 10.1097/01.ajp.0000210914.72794.bc

[23] Eide R. The relationship of pain sensation to cold pressor reactions and local cold habituation. Scand J Clin Lab Invest 1965;17:584–588. 5858750

[24] Eitner L Özgül Ö S Enax-Krumova EK Vollert J Maier C Höffken O. Conditioned pain modulation using painful cutaneous electrical stimulation or simply habituation? Eur J Pain 2018;22:1281–1290. 29573038 10.1002/ejp.1215

[25] Ellerbrock I Wiehler A Arndt M May A. Nocebo context modulates long-term habituation to heat pain and influences functional connectivity of the operculum. PAIN 2015;156:2222–2233. 26181304 10.1097/j.pain.0000000000000297

[26] Ernst M Lee MH Dworkin B Zaretsky HH. Pain perception decrement produced through repeated stimulation. PAIN 1986;26:221–231. 3763235 10.1016/0304-3959(86)90077-1

[27] Gács B Szolcsányi T Csathó Á. Opposite patterns of change in perception of imagined and physically induced pain over the course of repeated thermal stimulations. Eur J Pain 2017;21:1165–1172. 28230300 10.1002/ejp.1017

[28] Gallez A Albanese MC Rainville P Duncan GH. Attenuation of sensory and affective responses to heat pain: evidence for contralateral mechanisms. J Neurophysiol 2005;94:3509–3515. 16222074 10.1152/jn.01006.2004

[29] Garcia-Larrea L Frot M Valeriani M. Brain generators of laser-evoked potentials: from dipoles to functional significance. Neurophysiol Clin 2003;33:279–292. 14678842 10.1016/j.neucli.2003.10.008

[30] Goebel R Esposito F Formisano E. Analysis of functional image analysis contest (FIAC) data with brainvoyager QX: from single‐subject to cortically aligned group general linear model analysis and self‐organizing group independent component analysis. Hum Brain Mapp 2006;27:392–401. 16596654 10.1002/hbm.20249 PMC6871277

[31] Granovsky Y Anand P Nakae A Nascimento O Smith B Sprecher E Valls-Solé J. Normative data for Aδ contact heat evoked potentials in adult population: a multicenter study. PAIN 2016;157:1156–1163. 26907092 10.1097/j.pain.0000000000000495

[32] Greffrath W Baumgärtner U Treede RD. Peripheral and central components of habituation of heat pain perception and evoked potentials in humans. PAIN 2007;132:301–311. 17533117 10.1016/j.pain.2007.04.026

[33] Griffith CR. The organic effects of repeated bodily rotation. J Exp Psychol 1920;3:15.

[34] Groves PM Thompson RF. Habituation: a dual-process theory. Psychol Rev 1970;77:419. 4319167 10.1037/h0029810

[35] Haefeli J Kramer JL Blum J Curt A. Heterotopic and homotopic nociceptive conditioning stimulation: distinct effects of pain modulation. Eur J Pain 2014;18:1112–1119. 24443293 10.1002/j.1532-2149.2014.00454.x

[36] Hahn A Kranz GS Seidel E-M Sladky R Kraus C Küblböck M Pfabigan DM Hummer A Grahl A Ganger S. Comparing neural response to painful electrical stimulation with functional MRI at 3 and 7 T. Neuroimage 2013;82:336–343. 23769917 10.1016/j.neuroimage.2013.06.010

[37] Hardwicke TE Wagenmakers E-J. Reducing bias, increasing transparency and calibrating confidence with preregistration. Nat Hum Behav 2023;7:15–26. 36707644 10.1038/s41562-022-01497-2

[38] Hashmi JA Davis KD. Women experience greater heat pain adaptation and habituation than men. PAIN 2009;145:350–357. 19632779 10.1016/j.pain.2009.07.002

[39] Hashmi JA Davis KD. Effects of temperature on heat pain adaptation and habituation in men and women. PAIN 2010;151:737–743. 20926193 10.1016/j.pain.2010.08.046

[40] Hoeppli ME Nahman-Averbuch H Hinkle WA Leon E Peugh J Lopez-Sola M King CD Goldschneider KR Coghill RC. Dissociation between individual differences in self-reported pain intensity and underlying fMRI brain activation. Nat Commun 2022;13:3569. 35732637 10.1038/s41467-022-31039-3 PMC9218124

[41] Hüllemann P Mahn F Shao YQ Watfeh R Wasner G Binder A Baron R. Repetitive ipsilateral painful A-delta fibre stimuli induce bilateral LEP amplitude habituation. Eur J Pain 2013;17:1483–1490. 23716481 10.1002/j.1532-2149.2013.00335.x

[42] Hüllemann P Shao YQ Manthey G Binder A Baron R. Central habituation and distraction alter C-fibre-mediated laser-evoked potential amplitudes. Eur J Pain 2016;20:377–385. 26076052 10.1002/ejp.735

[43] Hüllemann P Watfeh R Shao YQ Nerdal A Binder A Baron R. Peripheral sensitization reduces laser-evoked potential habituation. Neurophysiol Clin 2015;45:457–467. 26602971 10.1016/j.neucli.2015.10.088

[44] Iannetti GD Hughes NP Lee MC Mouraux A. Determinants of laser-evoked EEG responses: pain perception or stimulus saliency? J Neurophysiol 2008;100:815–828. 18525021 10.1152/jn.00097.2008 PMC2525705

[45] Ibinson JW Small RH Algaze A Roberts CJ Clark DL Schmalbrock P. Functional magnetic resonance imaging studies of pain: an investigation of signal decay during and across sessions. J Am Soc Anesthesiologists 2004;101:960–969. 10.1097/00000542-200410000-00022 15448530

[46] Iwata K Tsuboi Y Yagi J Toda K Furukawa T Yoshimoto A Sumino R. Effect of interstimulus interval on perceived sensation and intradental nerve activity during thermal tooth stimulation in man. Brain Res 1994;635:211–216. 8173957 10.1016/0006-8993(94)91441-9

[47] Jepma M Jones M Wager TD. The dynamics of pain: evidence for simultaneous site-specific habituation and site-nonspecific sensitization in thermal pain. J Pain 2014;15:734–746. 24768695 10.1016/j.jpain.2014.02.010 PMC4083082

[48] Kassambara A. ggpubr: 'ggplot2' Based Publication Ready Plots, 2020. R package version 0.4.0

[49] Kersebaum D Fabig SC Sendel M Muntean AC Baron R Hullemann P. Revealing the time course of laser-evoked potential habituation by high temporal resolution analysis. Eur J Pain 2021;25:2112–2128. 34155707 10.1002/ejp.1823

[50] Klein T Magerl W Hopf H-C Sandkühler J Treede R-D. Perceptual correlates of nociceptive long-term potentiation and long-term depression in humans. J Neurosci 2004;24:964–971. 14749441 10.1523/JNEUROSCI.1222-03.2004 PMC6729815

[51] Kleinböhl D Trojan J Konrad C Hölzl R. Sensitization and habituation of AMH and C-fiber related percepts of repetitive radiant heat stimulation. Clin Neurophysiol 2006;117:118–130. 16256426 10.1016/j.clinph.2005.08.023

[52] Koenen LR Icenhour A Forkmann K Pasler A Theysohn N Forsting M Bingel U Elsenbruch S. Greater fear of visceral pain contributes to differences between visceral and somatic pain in healthy women. PAIN 2017;158:1599–1608. 28426553 10.1097/j.pain.0000000000000924

[53] Konorski J. Integrative activity of the brain. Chicago: University of Chicago Press, 1967.

[54] Lacadie CM Fulbright RK Rajeevan N Constable RT Papademetris X. More accurate Talairach coordinates for neuroimaging using non-linear registration. Neuroimage 2008;42:717–725. 18572418 10.1016/j.neuroimage.2008.04.240 PMC2603575

[55] LeBlanc J Potvin P. Studies on habituation to cold pain. Can J Physiol Pharmacol 1966;44:287–293. 5946564 10.1139/y66-033

[56] Loggia ML Jensen K Gollub RL Wasan AD Edwards RRK J. The catechol-O-methyltransferase (COMT) val158met polymorphism affects brain responses to repeated painful stimuli. PLoS One 2011;6:e27764. 22132136 10.1371/journal.pone.0027764 PMC3221673

[57] Lutz A McFarlin DR Perlman DM Salomons TV Davidson RJ. Altered anterior insula activation during anticipation and experience of painful stimuli in expert meditators. Neuroimage 2013;64:538–546. 23000783 10.1016/j.neuroimage.2012.09.030 PMC3787201

[58] Maeoka H Hiyamizu M Matsuo A Morioka S. The influence of repeated pain stimulation on the emotional aspect of pain: a preliminary study in healthy volunteers. J Pain Res 2015;8:431–436. 26229502 10.2147/JPR.S86732 PMC4516348

[59] Mancini F Pepe A Bernacchia A Di Stefano G Mouraux A Iannetti GD. Characterizing the short-term habituation of event-related evoked potentials. eNeuro 2018;5:14. 10.1523/ENEURO.0014-18.2018 PMC6162078 30280121

[60] Martucci KT Mackey SC. Neuroimaging of pain: human evidence and clinical relevance of central nervous system processes and modulation. Anesthesiology 2018;128:1241–1254. 29494401 10.1097/ALN.0000000000002137 PMC5953782

[61] Matre D Hu L Viken LA Hjelle IB Wigemyr M Knardahl S Sand T Nilsen KB. Experimental sleep restriction facilitates pain and electrically induced cortical responses. Sleep 2015;38:1607–1617. 26194577 10.5665/sleep.5058 PMC4576335

[62] McDiarmid TA Yu AJ Rankin CH. Habituation is more than learning to ignore: multiple mechanisms serve to facilitate shifts in behavioral strategy. BioEssays 2019;41:1900077. 10.1002/bies.201900077 31429094

[63] Mobascher A Brinkmeyer J Warbrick T Musso F Schlemper V Wittsack HJ Saleh A Schnitzler A Winterer G. Brain activation patterns underlying fast habituation to painful laser stimuli. Int J Psychophysiol 2010;75:16–24. 19833154 10.1016/j.ijpsycho.2009.10.008

[64] Mobascher A Brinkmeyer J Warbrick T Musso F Wittsack HJ Stoermer R Saleh A Schnitzler A Winterer G. Fluctuations in electrodermal activity reveal variations in single trial brain responses to painful laser stimuli - a fMRI/EEG study. Neuroimage 2009;44:1081–1092. 18848631 10.1016/j.neuroimage.2008.09.004

[65] Mouraux A Paepe ALD Marot E Plaghki L Iannetti GD Legrain V. Unmasking the obligatory components of nociceptive event-related brain potentials. J Neurophysiol 2013;110:2312–2324. 23966678 10.1152/jn.00137.2013

[66] Ødegård SS Omland PM Nilsen KB Stjern M Gravdahl GB Sand T. The effect of sleep restriction on laser evoked potentials, thermal sensory and pain thresholds and suprathreshold pain in healthy subjects. Clin Neurophysiol 2015;126:1979–1987. 25579466 10.1016/j.clinph.2014.12.011

[67] Papademetris X Jackowski MP Rajeevan N DiStasio M Okuda H Constable RT Staib LH. BioImage Suite: An integrated medical image analysis suite: An update. Insight J. 2006;209:1–8. PMC4213804 25364771

[68] Paul K Tik M Hahn A Sladky R Geissberger N Wirth EM Kranz GS Pfabigan DM Kraus C Lanzenberger R Lamm C Windischberger C. Give me a pain that I am used to: distinct habituation patterns to painful and non-painful stimulation. Scientific Rep 2021;11:10. 10.1038/s41598-021-01881-4 PMC8617189 34824311

[69] Pazzaglia C Testani E Giordano R Padua L Valeriani M. Expectation to feel more pain disrupts the habituation of laser-pain rating and laser-evoked potential amplitudes. Neuroscience 2016;333:244–251. 27461877 10.1016/j.neuroscience.2016.07.027

[70] Peckham GW Peckham EG. Some observations on the mental powers of spiders, J. Morphol 1887;2:383–419.

[71] Pedersen T. ggforce: Accelerating 'ggplot2', 2022. R package version 0.3.4

[72] Quiton RL Greenspan JD. Across-and within-session variability of ratings of painful contact heat stimuli. PAIN 2008;137:245–256. 17942227 10.1016/j.pain.2007.08.034 PMC5105332

[73] R Core Team. R: A Language and Environment for Statistical Computing. Vienna, Austria: R Foundation for Statistical Computing, 2022.

[74] Raja SN Carr DB Cohen M Finnerup NB Flor H Gibson S Keefe FJ Mogil JS Ringkamp M Sluka KA Song XJ Stevens B Sullivan MD Tutelman PR Ushida T Vader K. The revised International Association for the Study of Pain definition of pain: concepts, challenges, and compromises. PAIN 2020;161:1976–1982. 32694387 10.1097/j.pain.0000000000001939 PMC7680716

[75] Rankin CH Abrams T Barry RJ Bhatnagar S Clayton DF Colombo J Coppola G Geyer MA Glanzman DL Marsland S. Habituation revisited: an updated and revised description of the behavioral characteristics of habituation. Neurobiol Learn Mem 2009;92:135–138. 18854219 10.1016/j.nlm.2008.09.012 PMC2754195

[76] Rennefeld C Wiech K Schoell ED Lorenz J Bingel U. Habituation to pain: further support for a central component. PAIN 2010;148:503–508. 20097005 10.1016/j.pain.2009.12.014

[77] Riedl V Valet M Wöller A Sorg C Vogel D Sprenger T Boecker H Wohlschläger AM Tölle TR. Repeated pain induces adaptations of intrinsic brain activity to reflect past and predict future pain. Neuroimage 2011;57:206–213. 21514392 10.1016/j.neuroimage.2011.04.011

[78] Rodriguez-Raecke R Doganci B Breimhorst M Stankewitz A Büchel C Birklein F May A. Insular cortex activity is associated with effects of negative expectation on nociceptive long-term habituation. J Neurosci 2010;30:11363–11368. 20739557 10.1523/JNEUROSCI.2197-10.2010 PMC6633339

[79] Ronga I Valentini E Mouraux A Iannetti GD. Novelty is not enough: laser-evoked potentials are determined by stimulus saliency, not absolute novelty. J Neurophysiol 2013;109:692–701. 23136349 10.1152/jn.00464.2012 PMC3567386

[80] Rorden C Karnath HO Bonilha L. Improving lesion-symptom mapping. J Cogn Neurosci 2007;19:1081–1088. 17583985 10.1162/jocn.2007.19.7.1081

[81] Rosier EM Iadarola MJ Coghill RC. Reproducibility of pain measurement and pain perception. PAIN 2002;98:205–216. 12098633 10.1016/s0304-3959(02)00048-9

[82] RStudio Team. RStudio: Integrated Development Environment for R. Boston, MA: RStudio, PBC, 2022.

[83] Ruscheweyh R Emptmeyer K Putzer D Kropp P Marziniak M. Reproducibility of contact heat evoked potentials (CHEPs) over a 6 months interval. Clin Neurophysiol 2013;124:2242–2247. 23746497 10.1016/j.clinph.2013.05.003

[84] Santoro M Vollono C Pazzaglia C Di Sipio E Giordano R Padua L Arendt-Nielsen L Valeriani M. ZNRD1-AS and RP11-819C21.1 long non-coding RNA changes following painful laser stimulation correlate with laser-evoked potential amplitude and habituation in healthy subjects: a pilot study. Eur J Pain 2020;24:593–603. 31782860 10.1002/ejp.1511

[85] Scheuren PS Rosner J Curt A Hubli M. Pain-autonomic interaction: a surrogate marker of central sensitization. Eur J Pain 2020;24:2015–2026. 32794307 10.1002/ejp.1645

[86] Schmidt K Schunke O Forkmann K Bingel U. Enhanced short-term sensitization of facial compared with limb heat pain. J Pain 2015;16:781–790. 26043953 10.1016/j.jpain.2015.05.003

[87] Schuh-Hofer S Baumgartner U Treede RD. Effect of sleep deprivation on the electrophysiological signature of habituation to noxious laser stimuli. Eur J Pain 2015;19:1197–1209. 25851512 10.1002/ejp.698

[88] Slepian PM France CR Rhudy JL Himawan LK Güereca YM Kuhn BL Palit S. Behavioral inhibition and behavioral activation are related to habituation of nociceptive flexion reflex, but not pain ratings. J Pain 2017;18:349–358. 27919775 10.1016/j.jpain.2016.11.010

[89] Sokolov EN. Higher nervous activity and the problem of perception 1. In: Simon, B, editor. Psychology in the Soviet Union Ils 272. New York, NY: Routledge 1957. pp. 92–99.

[90] Sokolov EN. Neuronal models and the orienting reflex. In: Brazier, MAB, editor. The central nervous system and behaviour. New York, NY: Josiah Macy Jr. Foundation. pp. 187–276.

[91] Stancak A Yamamotova A Kulis IP Sekyra IV. Cardiovascular adjustments and pain during repeated cold pressor test. Clin Auton Res 1995;6:83–89. 10.1007/BF02291228 8726092

[92] Stankewitz A Valet M Schulz E Wöller A Sprenger T Vogel D Zimmer C Mühlau M Tölle TR. Pain sensitisers exhibit grey matter changes after repetitive pain exposure: a longitudinal voxel-based morphometry study. PAIN 2013;154:1732–1737. 23685021 10.1016/j.pain.2013.05.019

[93] Szikszay TM Adamczyk WM Levenez JLM Gouverneur P Luedtke K. Temporal properties of pain contrast enhancement using repetitive stimulation. Eur J Pain 2022;26:1437–1447. 35535976 10.1002/ejp.1971

[94] Thompson RF. Habituation: a history. Neurobiol Learn Mem 2009;92:127. 18703156 10.1016/j.nlm.2008.07.011 PMC2714193

[95] Thompson RF Spencer WA. Habituation: a model phenomenon for the study of neuronal substrates of behavior. Psychol Rev 1966;73:16. 5324565 10.1037/h0022681

[96] Torta DM Liang M Valentini E Mouraux A Iannetti GD. Dishabituation of laser-evoked EEG responses: dissecting the effect of certain and uncertain changes in stimulus spatial location. Exp Brain Res 2012;218:361–372. 22349498 10.1007/s00221-012-3019-6

[97] Treister R Eisenberg E Gershon E Haddad M Pud D. Factors affecting - and relationships between-different modes of endogenous pain modulation in healthy volunteers. Eur J Pain 2010;14:608–614. 19906552 10.1016/j.ejpain.2009.10.005

[98] Tricco AC Lillie E Zarin W O'Brien KK Colquhoun H Levac D Moher D Peters MD Horsley T Weeks L. PRISMA extension for scoping reviews (PRISMA-ScR): checklist and explanation. Ann Intern Med 2018;169:467–473. 30178033 10.7326/M18-0850

[99] Valentini E Torta DM Mouraux A Iannetti GD. Dishabituation of laser-evoked EEG responses: dissecting the effect of certain and uncertain changes in stimulus modality. J Cogn Neurosci 2011;23:2822–2837. 21265604 10.1162/jocn.2011.21609

[100] Valeriani M Rambaud L Mauguière F. Scalp topography and dipolar source modelling of potentials evoked by CO2 laser stimulation of the hand. Electroencephalography Clin Neurophysiology/Evoked Potentials Section 1996;100:343–353. 10.1016/0168-5597(96)95625-7 17441304

[101] van den Broeke E Crombez G Vlaeyen JW. Re-conceptualizing sensitization in pain: A case for a behavioural approach. PsychArchives 2023;1-7.

[102] van der Miesen M M Vossen CJ Joosten EA. Habituation to Pain in Patients with Chronic Pain: Clinical Implications and Future Directions. J Clin Med 2023;12:1–25. 10.3390/jcm12134305 PMC10342770 37445339

[103] Vossen CJ Vossen HG Marcus MA van Os J Lousberg R. Introducing the event related fixed interval area (ERFIA) multilevel technique: a method to analyze the complete epoch of event-related potentials at single trial level. PLoS One 2013;8:e79905. 24224018 10.1371/journal.pone.0079905 PMC3817110

[104] Vossen HGM van Breukelen G Hermens H van Os J Lousberg R. More potential in statistical analyses of event-related potentials: a mixed regression approach. Int J Methods Psychiatr Res 2011;20:e56–e68. 21812066 10.1002/mpr.348 PMC6878471

[105] Vossen HGM van Breukelen GJP van Os J Hermens HJ Lousberg R. Association between event-related potentials and pain ratings not as straightforward as often thought. J Psychophysiol 2011;25:18–25.

[106] Wagner AR. Habituation and Memory. In: Dickinson A, Boakes RA, editors. Mechanisms of learning and motivation: A memorial volume for Jerzy Konorski. Hillsdale, NJ: Lawrence Erlbaum Associates, 1979. pp. 53–82.

[107] Weissman‐Fogel I Granovsky Y Bar‐Shalita T. Sensory over‐responsiveness among healthy subjects is associated with a pronociceptive state. Pain Pract 2018;18:473–486. 28782305 10.1111/papr.12619

[108] Wickham H. ggplot2: Elegant Graphics for Data Analysis. New York: Springer-Verlag, 2016.

[109] Wilcox CE Mayer AR Teshiba TM Ling J Smith BW Wilcox GL Mullins PG. The subjective experience of pain: an FMRI study of percept-related models and functional connectivity. Pain Med 2015;16:2121–2133. 25989475 10.1111/pme.12785 PMC4653099

[110] Xu A Larsen B Baller EB Scott JC Sharma V Adebimpe A Satterthwaite TD. Convergent neural representations of experimentally-induced acute pain in healthy volunteers: A large-scale fMRI meta-analysis. Neurosci Biobehav Rev 2020;112:300–323. 31954149 10.1016/j.neubiorev.2020.01.004 PMC7755074

[111] Zbrozyna A Westwood D. Habituation and recovery of vascular responses in calf and forearm and of the level of pain sensation during the cold pressor test in man. Eur J Appl Physiol Occup Physiol 1990;61:106–111. 2289485 10.1007/BF00236702

