# Supplementary material for: Habituation to pain: self-report, electroencephalography, and functional magnetic resonance imaging in healthy individuals. A scoping review and future recommendations
Source: Pain. 2023 Oct 18;165(3):500–22. doi: 10.1097/j.pain.0000000000003052 (PMC10859850; doi:10.1097/j.pain.0000000000003052)
Supplement: Supplementary file 1 [file jop-165-500-s001.pdf]

Full search strategy for PubMed database

((("Chronic Pain"[Mesh] OR chronic Pain*[tiab] OR "Pain"[Mesh] OR pain*[tiab] OR "Pain Threshold"[Mesh] OR pain threshold*[tiab] OR ache*[tiab] OR physical suffering*[tiab] OR

arthrosis[tiab] OR arthropathy*[tiab] OR arthritis[tiab] OR cephalic syndrome*[tiab] OR contracture*[tiab] OR chondropath*[tiab] OR cephalgia*[tiab] OR cephalea*[tiab] OR cephalalgia*[tiab] OR "Colonic Diseases, Functional"[Mesh] OR colon disease*[tiab] OR colonic disease*[tiab] OR Crohn*[tiab] OR colitis[tiab] OR colon diverticulosis[tiab] OR colon fistula*[tiab] OR colorectal disease*[tiab] OR "Endometriosis"[Mesh] OR endometriosis[tiab] OR endometrioses[tiab] OR endometrioma[tiab] OR enthesopath*[tiab] OR fibrositis[tiab] OR fibromyalgia[tiab] OR "Fibromyalgia"[Mesh] OR functional colonic disease*[tiab] OR “Headache Disorders"[Mesh] OR headache*[tiab] OR ischialgia[tiab] OR irritable bowel syndrome*[tiab] OR irritable colon*[tiab] OR irritable colon syndrome*[tiab] OR IBD[tiab] OR inflammatory bowel disease*[tiab] OR migraine*[tiab] OR mononeuropath*[tiab] OR musculoskeletal disease*[tiab] OR myalgia[tiab] OR nerve fiber inflammation*[tiab] OR nerve compression*[tiab] OR nerve entrapment*[tiab] OR nervous entrapment*[tiab] OR nerve constriction*[tiab] OR nerve injur*[tiab] OR neuralg*[tiab] OR neuritis[tiab] OR neuropath*[tiab] OR osteoarthrosis[tiab] OR osteoarthritis[tiab] OR phantom limb*[tiab] OR "Peripheral Nervous System Diseases"[Mesh] OR peripheral nervous system Disease*[tiab] OR PNS disease*[tiab] OR peripheral nerve disease*[tiab] OR peripheral nervous disease*[tiab] OR peripheral nervous system Disorder*[tiab] OR PNS disorder*[tiab] OR peripheral nerve disorder*[tiab] OR peripheral nervous disorder*[tiab] OR polyneuritis[tiab] OR polyradiculoneuritis[tiab] OR polyneuropath*[tiab] OR radiculoneuritis[tiab] OR radiculopath*[tiab] OR rheumatic[tiab] OR rheumatism[tiab] OR Spondyloarthritis[tiab] OR spondylarthritis[tiab] OR spondylitis[tiab] OR Sciatica[tiab])))

AND ("Habituation, Psychophysiologic"[MeSH] OR habituat*[tiab] OR repeated stim*[tiab] OR repeated pain*[tiab] OR repeated noxious[tiab] OR repetitive pain*[tiab] OR repetitive noxious[tiab] OR repetitive stim*[tiab])

Supplementary Table 1. Peak coordinates of fMRI studies included for visualization.

Study	Timescale	Contrast	Region	MNI Coordinates (mm)		
				X	Y	Z
**Bauch et al., 2017**	Short-term	Interaction between intensity, drug and time (over two runs), habituation was found in these areas	Left postcentral gyrus	-36	-24	46
			Left MCC	-2	-14	48
**Becerra et al., 1999**	Short-term	Stimulus 1 + 2 > stimulus 3 + 4	Frontal gyrus (mid)	26	47	12
			Left Insula	-35	18	0
			Anterior cingulate gyrus	-1	12	33
			Posterior cingulate gyrus	-4	-42	39
			Frontal gyrus (medial)	-38	26	43
			Temporal gyrus (mid)	57	-48	-2
			Pre-central gyrus	-62	5	17
**Bingel et al., 2007**	Long-term	Day 1 > day 8	Right thalamus	0	-9	6
			Right anterior insula	42	9	12
			Right S2	63	-18	18
			Right putamen	27	6	-9
			Left anterior insula	-33	18	6
			Left S2	-48	-9	21
			Left Putamen	-21	9	-3
		Day 1 < day 8	Right sgACC	3	36	-12
			Left sgACC	-6	30	-9
**Ellerbrock et al., 2015**	Long-term	Day 1 > day 21 (including control and nocebo group)	Left anterior insula	-40	4	0
			Right anterior insula	38	0	10
			Right S2	62	-24	26
			Left S2	-36	0	18

		Day 1 < day 21 (including control and nocebo group)	Right rostral ACC	-6	16	-10
**Hahn et al., 2013**	Short-term	Run 1 > run 4 3T	Temporal pole: superior temporal gyrus	-58	14	-6
			Right insula	28	22	-16
			Left S2	-62	-18	14
			MCC	0	40	24
		Run 1 > run 4 7T	Right postcentral gyrus	54	-22	42
			Left supramarginal gyrus	-64	-24	42
			Left insula	-40	-8	12
			Cerebellum	4	-72	-12
			PAG	0	-38	-12
			Cuneus	-2	-98	26
			Superior occipital gyrus	18	-82	36
			Fusiform gyrus/cerebellum	40	-72	-16
			Middle occipital gyrus	34	-84	6
**Ibinson et al., 2004**	Short-term	Stimulus 1 > stimulus 4	Left ACC	-8	-10	46
			Left S1	-38	-30	50
**Mobascher et al., 2010**	Short-term	First half (30 stimuli) > second half (30 stimuli)	Right parietal operculum	54	-26	20
			Right supramarginal gyrus	62	-26	36
			Right insula	38	2	2
			Right postcentral gyrus	52	-20	34
			Right precentral gyrus	44	0	58
			Right (pre)frontal cortex	42	24	-6
			Left parietal operculum	-60	-38	22

			Left insula	-36	20	0
			Left supramarginal gyrus	-64	-22	22
			ACC/mid-cingulate	4	8	36
			Precuneus	14	-72	40
**Paul et al., 2021**	Short-term	Run 1 > run 4	Right IFG	38	26	-12
			Right S2	54	-20	22
			aMCC	0	18	28
			Left S2	-58	-13	16
			Left IFG	-32	20	-14

Note: The peak coordinates of Hahn et al. (2013) were kindly provided by the authors upon request. For this study, the AAL3 atlas (as implemented in MRICron, Rorden et al. (2007)) was used together with the article to list the corresponding brain region of the peak MNI coordinates.

Rorden, C., Karnath, H. O., & Bonilha, L. (2007). Improving lesion-symptom mapping. *J Cogn Neurosci, 19*(7), 1081-1088. https://doi.org/10.1162/jocn.2007.19.7.1081
